# Novel genetic associations with five aesthetic facial traits: A genome-wide association study in the Chinese population

**DOI:** 10.3389/fgene.2022.967684

**Published:** 2022-08-12

**Authors:** Peiqi Wang, Xinghan Sun, Qiang Miao, Hao Mi, Minyuan Cao, Shan Zhao, Yiyi Wang, Yang Shu, Wei Li, Heng Xu, Ding Bai, Yan Zhang

**Affiliations:** ^1^ State Key Laboratory of Oral Diseases & National Clinical Research Center for Oral Diseases, West China Hospital of Stomatology, Sichuan University, Chengdu, China; ^2^ State Key Laboratory of Biotherapy and Cancer Center, West China Hospital, Sichuan University, Chengdu, China; ^3^ Genomic & Phenomic Data Center, Chengdu 23Mofang Biotechnology Co., Ltd, Chengdu, China; ^4^ Department of Biobank, Chengdu 23Mofang Biotechnology Co., Ltd, Chengdu, China; ^5^ Department of Laboratory Medicine/Research Center of Clinical Laboratory Medicine, West China Hospital, Sichuan University, Chengdu, China; ^6^ Department of Dermatology, Rare Disease Center, West China Hospital, Sichuan University, Chengdu, China; ^7^ Lung Cancer Center, West China Hospital, Sichuan University, Chengdu, China; ^8^ State Key Laboratory of Biotherapy, Department of Thoracic Oncology, Cancer Center, West China Hospital, Sichuan University, Chengdu, China

**Keywords:** facial trait, aesthetics, genome-wide association study, genome-wide polygenic score, widow’s peak

## Abstract

**Background:** The aesthetic facial traits are closely related to life quality and strongly influenced by genetic factors, but the genetic predispositions in the Chinese population remain poorly understood.

**Methods:** A genome-wide association studies (GWAS) and subsequent validations were performed in 26,806 Chinese on five facial traits: widow’s peak, unibrow, double eyelid, earlobe attachment, and freckles. Functional annotation was performed based on the expression quantitative trait loci (eQTL) variants, genome-wide polygenic scores (GPSs) were developed to represent the combined polygenic effects, and single nucleotide polymorphism (SNP) heritability was presented to evaluate the contributions of the variants.

**Results:** In total, 21 genetic associations were identified, of which ten were novel: *GMDS-AS1* (rs4959669, *p* = 1.29 × 10^−49^) and *SPRED2* (rs13423753, *p* = 2.99 × 10^−14^) for widow’s peak, a previously unreported trait; *FARSB* (rs36015125, *p* = 1.96 × 10^−21^) for unibrow; *KIF26B* (rs7549180, *p* = 2.41 × 10^−15^), *CASC2* (rs79852633, *p* = 4.78 × 10^−11)^, *RPGRIP1L* (rs6499632, *p* = 9.15 × 10^−11^), and *PAX1* (rs147581439, *p* = 3.07 × 10^−8^) for double eyelid; *ZFHX3* (rs74030209, *p* = 9.77 × 10^−14^) and *LINC01107* (rs10211400, *p* = 6.25 × 10^−10^) for earlobe attachment; and *SPATA33* (rs35415928, *p* = 1.08 × 10^−8^) for freckles. Functionally, seven identified SNPs tag the missense variants and six may function as eQTLs. The combined polygenic effect of the associations was represented by GPSs and contributions of the variants were evaluated using SNP heritability.

**Conclusion:** These identifications may facilitate a better understanding of the genetic basis of features in the Chinese population and hopefully inspire further genetic research on facial development.

## 1 Introduction

Facial features exhibit a higher degree of variability than other physical features, thus making human faces unique and recognizable. Appearance variations impact quality of life, in most cases, from the perspective of aesthetics. Although sometimes acquired over the lifespan due to external factors, the variation is closely connected with the inherited complexity of facial morphogenesis (Weinberg et al., 2013; Cole et al., 2017). The correlation has been extensively researched in genetic studies and experimental animal models (Weinberg et al., 2019), and a thorough understanding of the genetic basis of specific facial traits provides insights into, for instance, the mechanisms of facial morphogenesis as well as biometrics and forensic science (Kayser and De Knijff, 2011; Claes, 2014; Sturm and Duffy, 2018).

Despite evidence accumulated to illustrate the association between facial traits and genetic variants (Liu et al., 2012; Huang et al., 2021), a considerable fraction remains to be discovered. Our study aimed at five aesthetic facial features: widow’s peak, unibrow, double eyelid, earlobe attachment, and freckles. To date, the correlated genetic factors involved in some of these traits have been studied. For instance, unibrow has been reported with associations in 2q36 near the *PAX3* gene (Adhikari et al., 2016). Regarding eyelid trait that has a pronounced level of variation in East Asians, genome-wide association studies (GWASs) have revealed *HOXD-MTX2* to be relevant to eyelid curvature in Koreans (Seongwon et al., 2018) and *EMX2* associated with eyelid folding in Japanese (Chihiro et al., 2018). Meanwhile, a large-scale multiethnic GWAS revealed multiple loci associated with earlobe attachment harboring several candidate genes (e.g. *MRPS22*, *EDAR*, and *PAX9*) (Shaffer et al., 2017). Moreover, several variants of pigmentary genes, such as *BNC2*, *IRF4*, and *MC1R*, have been identified by recent studies, especially in Caucasians (Maarten et al., 2001; Eriksson et al., 2010; Jacobs et al., 2015; Kim et al., 2022), while only a few studies have been performed in Asians, mainly in Japanese and Korean population (Chihiro et al., 2018; Shido et al., 2019; Shin et al., 2021). In spite of the previous findings, the genetic background of these facial traits remains far from fully understood, especially in the Chinese population. Besides, although long been understood to have a genetic basis, genetic predispositions to widow’s peak, an important aesthetic trait, have not been reported.

**FIGURE 1:**
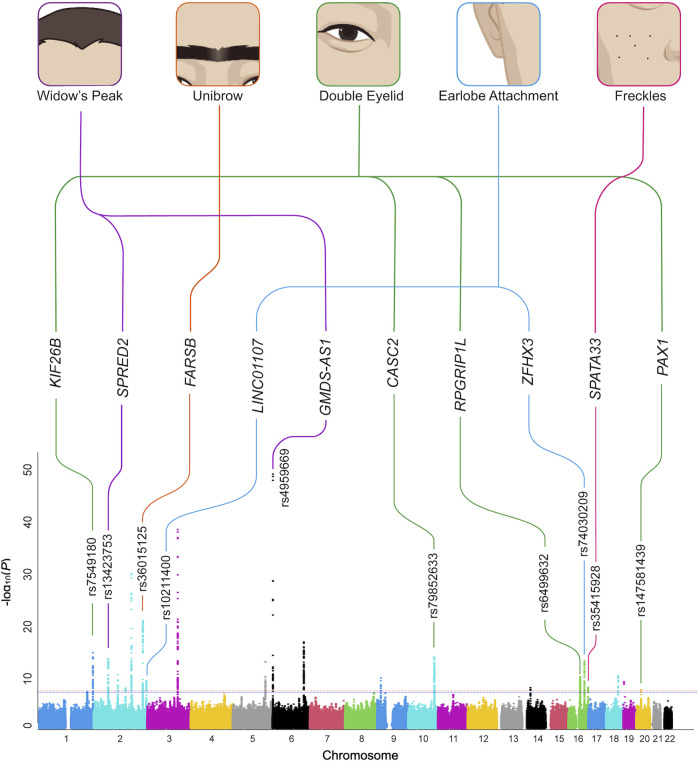
Overview of the GWAS results. The five aesthetic facial traits studied in the Chinese study sample (top) are connected with the candidate genes identified in regions with novel genome-wide significant associations. The GWAS results of the five traits were summarized on a single composite Manhattan plot (bottom). The rs ID of the SNP with the smallest *p*-value at the top of each association peak is given. GWAS, genome-wide association study. SNP, single nucleotide polymorphism.

Therefore, we performed a large-scale GWAS on the Chinese population to gain insights into genetic variants contributing to the five aesthetic facial traits (widow’s peak, unibrow, double eyelid, earlobe attachment, and freckles). Functional annotation of the genome-wide significant single nucleotide polymorphisms (SNPs) was performed based on the expression quantitative trait loci (eQTL) variants and genome-wide polygenic scores (GPSs) were subsequently developed to represent the combined polygenic effects in these five traits (Supplementary Figure S1). In total, 21 associations were identified and ten of them were novel (Figure 1). Specifically, to our knowledge, this is the first genetic report of widow’s peak, identifying *GMDS-AS1* (rs4959669, *p =* 1.29 × 10^−49^) and *SPRED2* (rs13423753, *p =* 2.99 × 10^−14^) as genome-wide significant associations in the Chinese population. The other novel associations included *FARSB* (rs36015125, *p =* 1.96 × 10^−21^) for unibrow; *KIF26B* (rs7549180, *p =* 2.41 × 10^−15^), *CASC2* (rs79852633, *p =* 4.78 × 10^−11^), *RPGRIP1L* (rs6499632, *p =* 9.15 × 10^−11^), and *PAX1* (rs147581439, *p =* 3.07 × 10^−8^) for double eyelid; *ZFHX3* (rs74030209, *p =* 9.77 × 10^−14^) and *LINC01107* (rs10211400, *p =* 6.25 × 10^−10^) for earlobe attachment; and *SPATA33* (rs35415928, *p =* 1.08 × 10^−8^) for freckles. This study was expected to facilitate a better understanding of the genetic basis of the facial features and inspire further research on the biological functions of the relevant genes.

## 2 Materials and methods

### 2.1 Subject, sample, and phenotypes

Subjects were voluntarily enrolled in the study and filled out the questionnaires designed by West China Hospital, Sichuan University. Questionnaires soliciting trait information including “widow’s peak,” “unibrow,” “double eyelid,” “earlobe attachment,” and “freckles,” were collected (Supplementary Methods). Subsequently, phenotypical data were filtered and merged on the grounds of the verification questions to ensure the authenticity and accuracy of the preprocessed data. The study was approved by the local ethics committee [West China Hospital, Sichuan University, approval no. 2017(241)] and all participants signed an electronic informed consent form. Methods were performed following the relevant guidelines and regulations.

### 2.2 DNA extraction and genotyping

Each participant donated 2 ml of their saliva into a sample tube which was later sent to the laboratory to extract DNA, and DNA quality was determined by examining the OD260/OD280 ratio and integrity in agarose gels. Due to the long time span of the project, samples were randomly genotyped with one of the three highly correlated versions of chip arrays – Mofang v1.0, Mofang v2.0, and Mofang v2.1, which were all Affymetrix Axiom Precision Medicine Research Array (PMRA)-based high-throughput SNP chip arrays (Affymetrix, Santa Clara, CA, United States).

### 2.3 Quality controls

To control the genotyping quality, QCs were performed at both the individual and SNP levels: 1) SNP with genotype call rate (CR) below 0.98, 2) individual CR below 0.98, 3) gender inconsistencies, 4) number of alleles >2, 5) minor allele frequency (MAF) below 0.01 (Supplementary Table S1), 6) deviation from Hardy–Weinberg equilibrium (*p*-value < 1 × 10^−6^), 7) outliers ±3 SD from the samples’ heterozygosity rate, 8) individuals with cryptic relatedness, 9) outliers from multidimensional scaling (MDS) analysis (Xu et al., 2015a; Xu et al., 2015b; Qian et al., 2019; Zhang et al., 2019; Hao et al., 2021; Hertz et al., 2021) (Supplementary Methods; Supplementary Figure S1).

### 2.4 Genome-wide association study

For each of the five traits, 80% of the samples were randomly selected to perform GWASs as the discovery set, and the rest 20% were used for validation (Supplementary Table S2). Additional QC was further performed before the association analyses: inclusion of SNPs with CR 0.98 and MAF ≥0.01, removal of heterozygosity outliers, removal of individuals with cryptic relatedness and population structure outliers. The genotype frequency between cases and controls was compared with sex, age, and five top principal components (PCs) as covariates, by the logistic regression model using PLINK v1.90b5.4 (Supplementary Methods) (Chang et al., 2015).

### 2.5 Functional annotation

The genome-wide SNPs were subjected to HaploReg database (https://pubs.broadinstitute.org/mammals/haploreg/haploreg.php) (Ward and Kellis, 2012), WashU EpiGenome Browser (http://epigenomegateway.wustl.edu/browser/) (Zhou et al., 2015) and Genotype-Tissue Expression (GTEx) dataset (https://gtexportal.org/home/) (GTEx Consortium, 2015) for functional annotation.

### 2.6 Construction of the GPSs

28 candidate GPSs based on a pruning and thresholding (P-T) method were derived for each trait using the GWAS discovery set and discovery GWAS summary statistics from the previous step. The best scores, defined by the maximal area under curve (AUC), were applied to the validation set with 20% samples to generate a polygenic score for each individual. The individuals were binned into 20 groups according to the GPS quantile and the prevalence of each trait (Supplementary Methods) (Khera et al., 2018).

### 2.7 Estimation of SNP heritability

To evaluate the contribution of the variants to heritability, SNP heritability of the five traits was estimated based on the GWAS summary statistics using linkage disequilibrium score regression analysis (LDSC) (Bulik-Sullivan et al., 2015).

## 3 Results

In this study, a total of 26,806 Chinese volunteers who passed QC were enrolled to investigate the associations between genetic variants and the five facial traits: widow’s peak (*N* = 11,946), unibrow (*N* = 7,254), double eyelid (*N* = 7,473), earlobe attachment (*N* = 9,977), and freckles (*N* = 8,251). All volunteers had phenotype information of one or more traits. The discovery and validation sets for each trait were randomly drawn respectively (Supplementary Table S2; Supplementary Figure S1). GWAS was performed for each trait, showing no obvious inflation (Supplementary Figure S2). Associations of genome-wide significant signals in the discovery sets with a predisposition to each trait were estimated in the respective validation sets to verify their reliability (Table 1; Supplementary Table S3).

**TABLE 1:** Summary of previously unreported genome-wide significant loci.

Region	SNP ID	Gene(s)	Alleles	OR [95% CI] (discovery)	*p*-value (discovery)	OR (validation)	*p*-value (validation)
Widow’s peak							
6p25.2	rs4959669	*GMDS-AS1*, *LINC01600*	T > C	0.49 [0.44–0.54]	1.29 × 10^−49^	0.50	3.87 × 10^−14^
2p14	rs13423753	*SPRED2*, *MIR4778*	G > A	0.78 [0.73–0.83]	2.99 × 10^−14^	0.80	5.65 × 10^−4^
Unibrow							
2q36.1	rs36015125	*FARSB*	C > G	0.69 [0.63–0.74]	1.96 × 10^−21^	0.73	6.76 × 10^−5^
Double Eyelid							
1q44	rs7549180	*KIF26B*	C > A	1.59 [1.42–1.78]	2.41 × 10^−15^	1.31	2.12 × 10^−2^
10q26.11	rs79852633	*CASC2*	G > A	1.35 [1.30–1.63]	4.78 × 10^−11^	1.59	5.82 × 10^−5^
16q12.2	rs6499632	*RPGRIP1L*	T > C	1.30 [1.20–1.40]	9.15 × 10^−11^	0.80	5.16 × 10^−3^
20p11.22	rs147581439	*PAX1*, *LINC01432*	G > C	2.06 [1.60–2.66]	3.07 × 10^−8^	2.00	6.81 × 10^−3^
Earlobe Attachment							
16q22.3	rs74030209	*ZFHX3*	C > T	0.77 [0.72–0.82]	9.77 × 10^−14^	0.85	1.83 × 10^−2^
2q37.3	rs10211400	*LINC01107*	G > T	0.75 [0.69–0.82]	6.25 × 10^−10^	0.73	5.75 × 10^−4^
Freckles							
16q24.3	rs35415928	*SPATA33*	C > T	1.43 [1.26–1.61]	1.08 × 10^−8^	1.34	1.57 × 10^−2^

### 3.1 Association analyses of the five facial traits

#### 3.1.1 Widow’s peak

**FIGURE 2:**
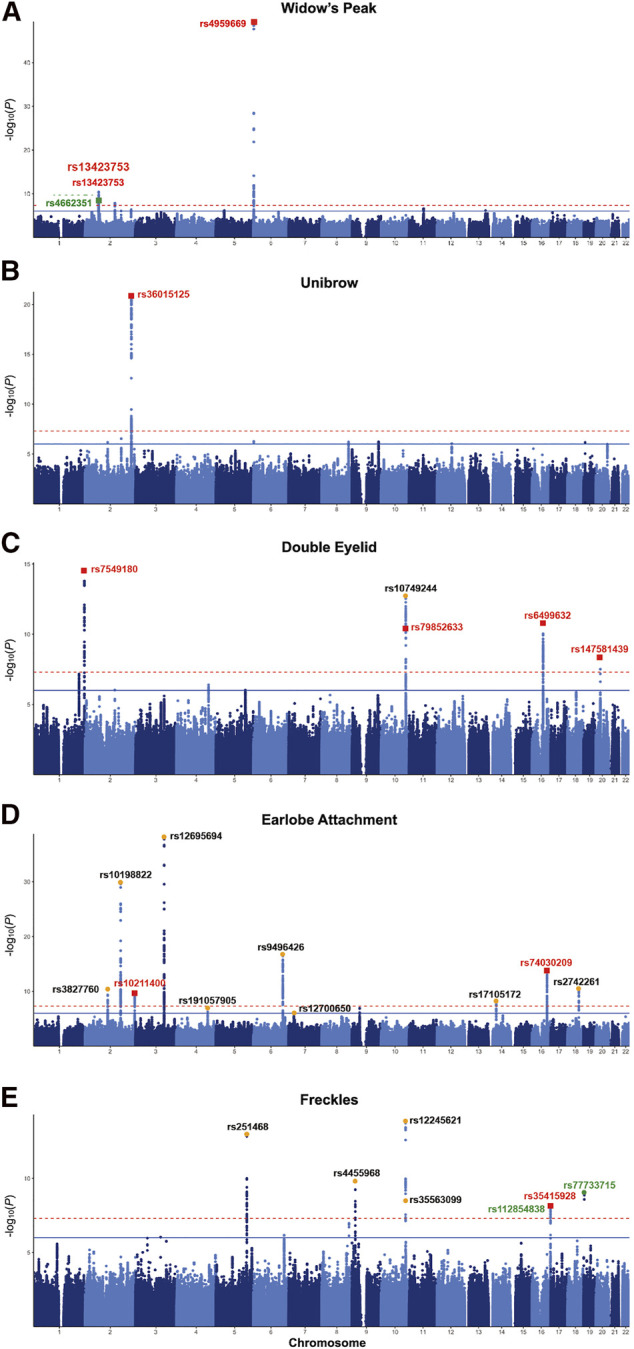
Manhattan plots of the discovery GWAS. Manhattan plot for **(A)** widow’s peak; **(B)** unibrow; **(C)** double eyelid; **(D)** earlobe attachment; **(E)** freckles. Bonferroni corrected threshold and candidate threshold correspond to 7.30 and 5.30, respectively, with regard to −log_10_ (*P*). Previously unreported SNPs are marked RED, previously reported SNPs are dotted ORANGE, and the SNPs failing validation are marked GREEN. GWAS, genome-wide association study. SNP, single nucleotide polymorphism.

**FIGURE 3:**
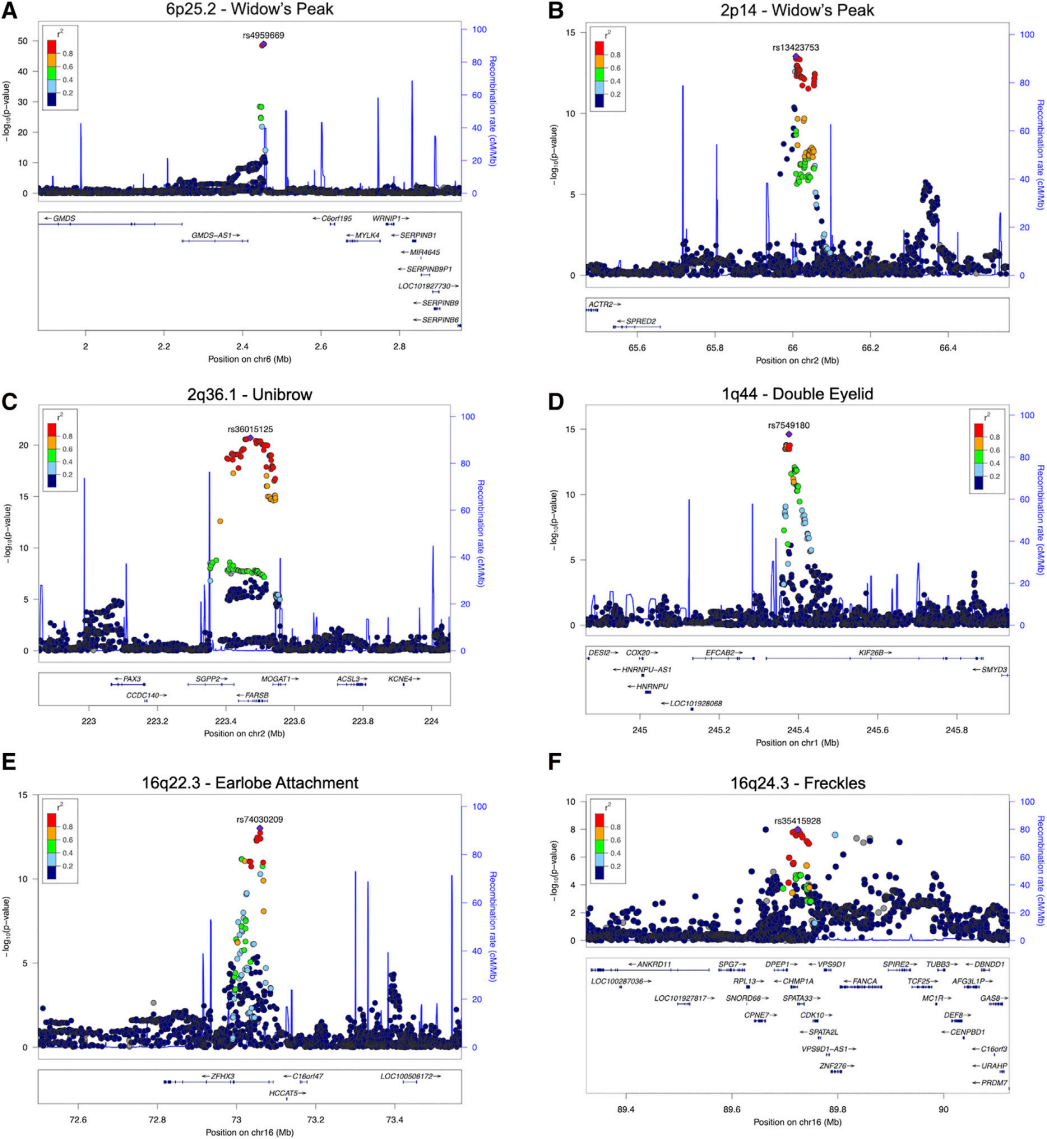
Regional association plots for eight regions with novel SNPs showing genome-wide significant associations with the five facial traits. Two novel associations for widow’s peak and novel associations with the smallest *p*-values for unibow, double, eyelid, earlobe attachment, and freckles are shown **(A)** and **(B)**, Regional association plot for **(A)** 6p25.2 and **(B)** 2p14 with novel SNP showing genome-wide significant association with widow’s peak; **(C)** regional association plot for 2q36.1 showing association with unibrow; **(D)** regional association plot for 1q44 showing association with double eyelid; **(E)** regional association plot for 16q22.3 showing association with earlobe attachment; **(F)** regional association plot for 16q24.3 showing association with freckles. SNP, single nucleotide polymorphism.

Although widow’s peak is regarded as a genetic heritable phenotypic pattern (Rassman et al., 2013; Kyriakou et al., 2021), genetic study of this trait is still lacking. In the present study, three loci reached genome-wide significance (*p*-value < 5 × 10^−8^) in the discovery cohort, including the strongest signals at 6p25.2 downstream *GMDS-AS1* (top SNP: rs4959669, *p =* 1.29 × 10^−49^), followed by 2p14 downstream *SPRED2* (top SNP: rs13423753, *p =* 3.0 × 10^−14^) and 2q22.3 downstream *ARHGAP15* (top SNP: rs4662351, *p =* 1.42 × 10^−8^) (Table 1; Figure 2A, Figures 3A,B). However, associations could only be reproduced for rs4959669 and rs13423753, but not for rs4662351 (Supplementary Table S3).

#### 3.1.2 Unibrow

As for unibrow, the only previously reported significant association signal, rs2218065 at 2q36.1 (Adhikari et al., 2016), could not be validated in our cohort (*p* = 3.11 × 10^−3^). Instead, we identified a locus 300–500 kb downstream of rs2218065 with the top signal at *FARSB* rs36015125 (*p =* 1.96 × 10^−21^) (Figure 2B, Figure 3C) and rs36015125 was not in linkage disequilibrium (LD) with rs2218065 (*r* ^2<^0.2).

#### 3.1.3 Double eyelid

Concerning double eyelid, five genetic loci were genome-wide significant and validated in the validation set**,** four of which have not been reported to our knowledge (Figure 2C). The loci at 10q26.11 replicated signals reported in Japanese women (Chihiro et al., 2018) with the top hit at rs10749244 near *EMX2* and *RAB11FIP2* (*p =* 1.96 × 10^−13^), in high LD with the reported variant rs1415425 (*r* ^2^ = 0.97) (Supplementary Figure S3D). A novel locus ∼500 kb downstream of the reported one, overlapping the long noncoding RNA (lncRNA) gene *CASC2* (top SNP: rs79852633, *p =* 4.78 × 10^−11^) exhibited an independent association (Supplementary Figure S3A). The other three unreported genome-wide significant loci overlapped *KIF26B* (1q44, top SNP: rs7549180, *p =* 5.75 × 10^−39^), *RPGRIP1L* (16q12.2, top SNP: rs6499632, *p =* 9.2 × 10^−11^), and *PAX1/LINC01432* (20p11.22, top SNP: rs147581439, *p =* 3.07 × 10^−8^), respectively (Figure 3D; Supplementary Figure S3).

#### 3.1.4 Earlobe attachment

For earlobe attachment, eight loci reached genome-wide significance in the discovery stage and were validated in a validation set (Figure 2D; Supplementary Table S3). Among them, two loci were novel to our knowledge, including a series of variants at 16q22.3 (top SNP: rs74030209, *p =* 9.8 × 10^−14^) and 2q37.3 (top SNP: rs10211400, *p =* 6.3 × 10^−10^) (Table 1; Figure 3E; Supplementary Figure S4A). The other six have been either previously reported or in LD with the reported SNPs in other ethnic populations (Supplementary Table S3; Supplementary Figure S4).

#### 3.1.5 Freckles

In pursuit of genetic associations with freckles, seven genome-wide significant loci were identified in the discovery stage, while only five passed validation (Figure 2E; Supplementary Table S3), among which only one has not been previously reported (16q24.3, *SPATA33*, top SNP: rs35415928, *p =* 1.1 × 10^−8^) (Table 1; Figure 3F). The other significant loci that passed validation overlapped *HSPA12A* (10q25.3), *PPARGC1B* (5q32), *BNC2* (9p22), and *EMX2*/*RAB11FIP2* (10q26.11) (Supplementary Figure S5).

### 3.2 The possible impact of the genome-wide significant associations

Functionally, the variants identified by GWAS may impact the corresponding phenotype by either altering the amino acids or regulating the expression of their nearby genes (Moriyama et al., 2016; Zhu et al., 2018; Tam et al., 2019; Moriyama et al., 2021). In this study, almost all the genome-wide significant variants are located in noncoding regions, except for rs3827760, a missense mutation point of the *EDAR* gene (Supplementary Table S3).

Meanwhile, seven variants are in LD (*r* ^2^ ≥ 0.2) with the coding variants based on LD calculations using 1,000 Genome Project data according to the HaploReg database (Supplementary Table S4) (Ward and Kellis, 2012). Therefore, we consider that the significant variants might mainly function by affecting gene expressions. Altogether, six of the genome-wide significant SNPs have GTEx eQTL associations (*p* < 1 × 10^−4^) in skin tissue and cultured fibroblasts that are of potential relevance to the five facial traits (Supplementary Table S5) (GTEx Consortium, 2015). Some of the SNPs show associations with only one gene. For instance, the previously unreported double eyelid-associated SNP rs6499632 is in LD with a missense variant of *RPGRIP1L* (*r* ^2^ = 0.26) and is a strong eQTL for *RP11-36I17.2* expression in cultured fibroblasts (Supplementary Table S4; Supplementary Figure S6). Meanwhile, some SNPs may have a tissue-specific eQTL association with multiple genes. The novel freckles-associated variant rs35415928 in *SPATA33* serves as a strong eQTL for several genes in both skin tissue (sun-exposed and no-sun-exposed) and fibroblasts, showing the strongest association with *DBNDD1* (Supplementary Figure S7), a gene involved in tanning ability (Nan et al., 2009) and squamous cell carcinoma (Asgari et al., 2016). Unibrow-associated *FARSB* rs36015125 is an eQTL for *RP11-16P6.1*, *SGPP2*, and *FARSB* expression in skin tissues (sun-exposed and no-sun-exposed) and cultured fibroblasts (Supplementary Figure S6), but information regarding these genes’ function in unibrow or hair appearance is unavailable.

### 3.3 Genome-wide polygenic score analysis

**FIGURE 4:**
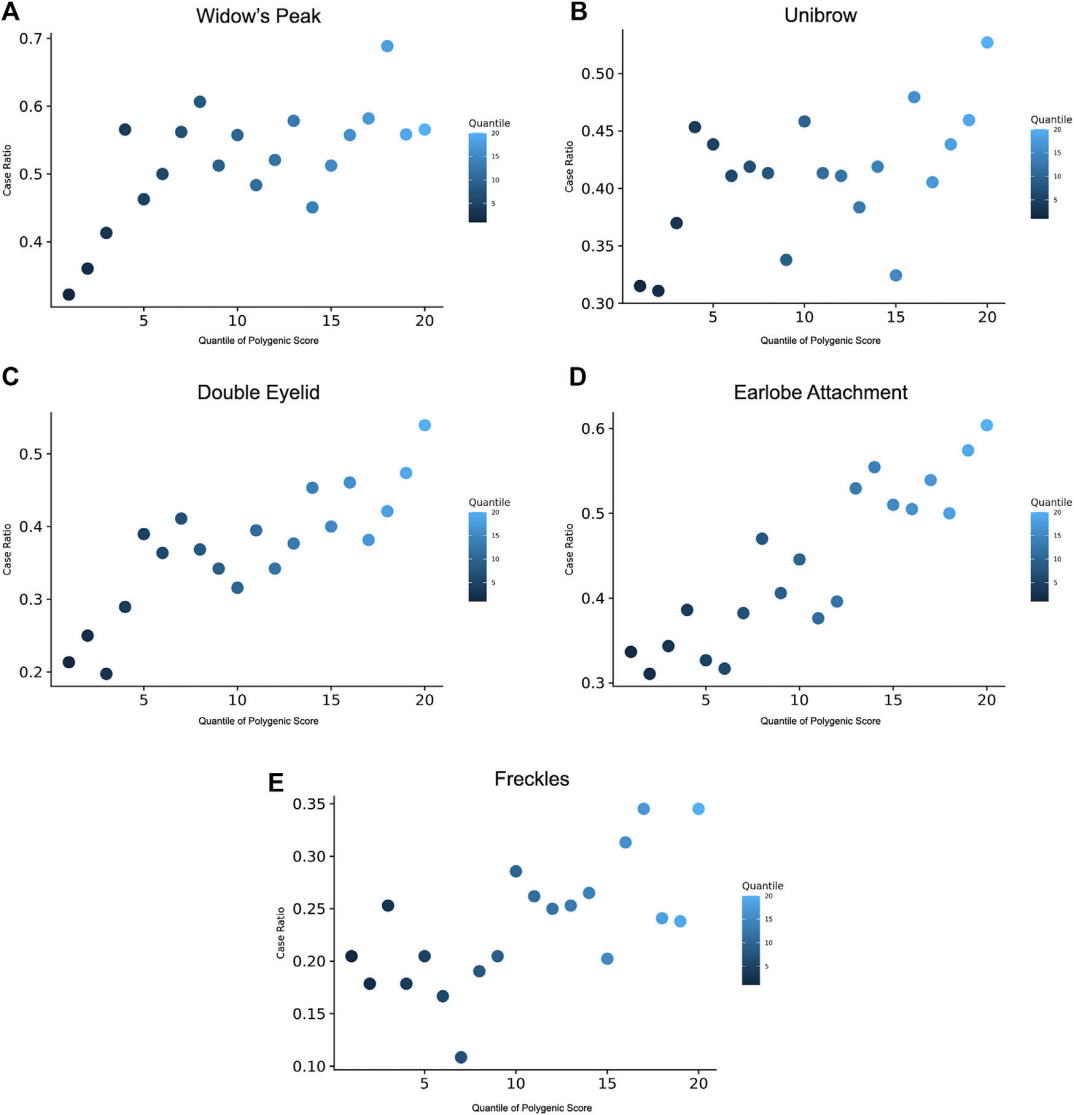
Prevalence of the traits according to the GPS quantile. 20 groups of the validation were derived based on the percentile of the GPS. Prevalence of phenotype displayed for the risk of **(A)** widow’s peak, **(B)** unibrow, **(C)** double eyelid, **(D)** earlobe attachment, and **(E)** freckles, within each quantile. GPS, genome-wide polygenic score.

GPS was constructed to manifest the genomic polygenic effect. For each trait, we derived 28 GPS predictors based on a P-T method from the discovery GWAS summary statistics and selected one best predictor defined by the maximal AUC in the discovery set (Supplementary Table S6). Taking widow’s peak as an example, the AUCs of the predictors ranged from 0.563 to 0.598 and reached the maximum when *p* = 1 × 10^−5^ and *r* ^2^ = 0.6 (Supplementary Table S6). Afterward, polygenic scores were generated in the validation set. Across the population, GPS was distributed with the empirical risk of the traits, showing a generally rising trend from 0.322 in the lowest quantile to 0.566 in the highest quantile (Figure 4A). Odds ratios (ORs) based on the quantile were given (Supplementary Table S7). Likewise, GPSs with the best performance were selected in the other four traits, and phenotype prevalence according to GPS was generated (Figures 4B–E). AUC reached maximum values of 0.594, 0.665, 0.657, and 0.625 in unibrow (*p =* 1 × 10^−5^, *r* ^2^ = 0.4), double eyelid (*p =* 1 × 10^−4^, *r* ^2^ = 0.2 or 0.8), earlobe attachment (*p =* 1 × 10^−5^, *r* ^2^ = 0.4), and freckles (*p =* 1 × 10^−7^, *r* ^2^ = 0.4), respectively (Supplementary Table S6).

### 3.4 SNP heritability of each trait

SNP heritability (*h* ^2^) using LDSC for the five traits was presented. The highest *h* ^2^ was seen for double eyelid (*h* ^2^ = 0.4487, standard error [SE] = 0.0765), and the *h* ^2^ for widow’s peak, unibrow, earlobe attachment, and freckles were estimated to be 0.3046 (SE = 0.0591), 0.433 (SE = 0.0881), 0.2443 (SE = 0.0765), and 0.1431 (SE = 0.0733), respectively.

## 4 Discussion

It is important to understand the complicated genetic background of the facial traits since it may facilitate further understanding of the basic mechanism of facial development. It becomes even more significant upon the notion that, while some facial traits only represent nonsyndromic conditions, some can be clinical manifestations of certain syndromes.

To our knowledge, we are the first to provide public GWAS results on widow’s peak, presenting two associated loci. The strongest signal was at 6p25.2 (rs4959669), near RNA genes *GMDS-AS1* and *LINC01600*, but further studies are needed to verify how the genes and their variants contribute to hairline morphology. The other association (rs13423753) occurred at 2p14 near *SPRED2*. Of potential relevance, some other members of the SPRED family have been reported to activate MAPK cascade (Nonami et al., 2004) which is implicated in hair follicle cell development (Yoon et al., 2011). Moreover, widow’s peak sometimes manifests as a symptom of certain syndromes, such as Donnai-Barrow syndrome (Longoni et al., 1993; Khalifa et al., 2015), Waardenburg syndrome type 1 (WS1), and Aarskog syndrome (Pingault et al., 2010), but variants related to these syndromes did not reach genome-wide significance in our study, probably due to the rare syndromic incidence and the difference among populations. It is important to take into consideration rare genetic-based disorders and diseases when discussing variant and phenotype association. Noteworthily, 23andMe Co. attempted to identify significant variants for unibrow and widow’s peak. According to the regional plot released on their website (https://medical.23andme.com/), associated loci for unibrow existed on several chromosomes, while significant variants for widow’s peak were located at 2q and 6p. Since detailed information from 23andMe Co.’s research is restrained, associations in our present GWAS provide novel insights for the public.

Unibrow is also related to attractiveness in many cultures. As far as we know, the only published associations for unibrow were in 2q36 with the lead SNP of rs2218065 near the *PAX3* gene, which was not validated in the present study (Adhikari et al., 2016). *PAX3* is a key transcription factor that guides the normal development of neural crest derivatives (Sang et al., 2012), and its mutations have been shown to cause WS1, 85% of which has manifestations including unibrow (Pingault et al., 2010). Of potential relevance, the PAX3 locus has previously been shown to control the location of “nasion,” the point at the middle of two eyebrows (Liu et al., 2012; Paternoster et al., 2012). In the present study, unlinked significant signals occurred near *PAX3* at 2q36.1, overlapping with the *FARSB* gene, a member of the *ARS* class IIc subfamily (Rodova et al., 1999). Other *ARS* members such as *KARS*, *CARS*, and *TARS* have been associated with hair phenotype (Santos-Cortez et al., 2013; Theil et al., 2019), suggesting a potential connection between *FARSB* and hair/brow development.

Interestingly, although East Asians are genetically closely-related, the present-day populations from different countries may have distinct genetic makeup (Wang et al., 2018), as seen in the pursuit of eyelid-associated variants. Among the five double eyelid-associated variants identified in our research, only rs10749244 at 10q26.11 is in high LD with previously reported rs1415425 (*r* ^2^ = 0.97) in Japanese (Chihiro et al., 2018). Among the four novel signals, the strongest association was observed for *KIF26B* rs7549180. In light of findings of *Kif26b* in the development of face (Marikawa et al., 2004), our results may suggest a regulatory role of *KIF26B* in the development of facial structure and concomitant upper eyelid differences. Functionally, *RPGRIP1L* rs6499632 serves as a strong GTEx eQTL for *RPGRIP1L* in cultured fibroblasts. The gene has been suggested to be involved in mechanisms such as craniofacial development, patterning of the limbs, and formation of the left-right axis (Delous et al., 2007). Another novel association (top SNP: rs147581439) overlapped with *PAX1*, a member of the *PAX* transcription factor family that plays a critical role during fetal development. Specifically, *PAX1* functions in pattern formation during embryogenesis (Wallin et al., 1994), and a missense mutation in *PAX1* has been shown to cause autosomal recessive Oto-Facio-Cervical syndrome, a disorder characterized by markedly skeletal and facial abnormalities (Pohl et al., 2013).

Regarding earlobe attachment, we identified two novel associations. One (rs74030209) is an intron point of *ZFHX3*, in LD with mutation points of the gene (Supplementary Table S4). *ZFHX3* is of potential relevance to ear development since it is involved in myogenic control by modulating myoblast differentiation (Berry et al., 2001), lack of which has been found to influence organogenesis in the inner ear phenotype (Rot et al., 2017). The other (rs10211400) is at the noncoding RNA (ncRNA) *LINC01107*. As some ncRNAs are correlated with nearby gene expression (Cabili et al., 2011; Guil and Esteller, 2012), the variant has a chance to be related to the regulation of *TWIST2* 314 kb downstream. Mutations of *TWIST2* have been associated with ectodermal dysplasia, such as Ablepharon-Macrostomia syndrome and Barber-Say syndrome (Marchegiani et al., 2015) whose manifestations include dysmorphic ears. Further studies are still needed to verify the conjecture. Besides these two unreported variants, our results of the attached earlobe mostly replicated the previous findings in diverse cohorts (Dutta and Ganguly, 1965; Adhikari et al., 2015; Shaffer et al., 2017). For instance, the strongest association was seen for the intergenic SNP rs12695694 near *MRPS22*, showing a strong GTEx eQTL association with *MRPS22* expression in cultured fibroblasts. Mutations of *MRPS22* have been previously implicated in earlobe size in Latin Americans and in lobe attachment in multiple cohorts (Adhikari et al., 2015; Shaffer et al., 2017), and relatively, a homozygous mutation in *MRPS22* has been reported to lead to oxidative phosphorylation system deficiency, which may manifest as dysmorphic features including low implanted posteriorly rotated ears (Smits et al., 2011). Meanwhile, the previously reported *EDAR* exonic variant rs3827760 (Bryk et al., 2008; Mou et al., 2008) also reached genome-wide significance in the present study. *EDAR* is involved in the prenatal development of ectoderm (Mikkola, 2009), and its deficiency has been suggested to result in abnormally shaped ears in mice (Adhikari et al., 2015).

Despite that pigmentary traits could be induced by extrinsic factors such as sun exposure, genetic predisposition has been suggested among different populations (Jacobs et al., 2015; Crawford et al., 2017; Chihiro et al., 2018; Adhikari et al., 2019; Shin et al., 2021). This may be because pigmentation is mainly contributed by a complicated process of melanin synthesis which is tightly associated with multiple genetic variants, and response after sun exposure is also genetically controlled (Nan et al., 2009; Shido et al., 2019). The freckles-associated variants identified in the present study were highly consistent with findings from Japanese and Korean cohorts (Chihiro et al., 2018; Shin et al., 2021), presumably due to the shared genetic backgrounds of East Asian populations. The only novel variant was *SPATA33* rs35415928. *SPATA33* has long been associated with facial pigmentation (Jacobs et al., 2015), cutaneous squamous cell carcinoma (Asgari et al., 2016), and melanoma (Fang et al., 2019). Closely downstream of the associations also lies the well-defined freckles-associated gene *MC1R.* (Maarten et al., 2001; Sulem et al., 2008; Eriksson et al., 2010). Among the identified associations, *BNC2* has been identified in Europeans (Jacobs et al., 2015). The top signal within *BNC2* (rs4455968) is in high LD with rs16935073 (*r* ^2^ = 0.94) and rs10816035 (*r* ^2^ = 0.83) that have been associated with pigmentary traits or tanning ability in Koreans (Shin et al., 2021) and Japanese (Chihiro et al., 2018; Shido et al., 2019). Another significant association existed in 10q25.3, led by *HSPA12A* rs12245621 which is in LD with the reported variant rs12259842 (*r* ^2^ = 0.76) (Chihiro et al., 2018). *HSPA12A* is affiliated to *HSP70* family whose members (*HSP70* and *HSP47*) are expressed in the dermis and epidermis following laser irradiation, which has been related to pigmentation (Sajjadi et al., 2013). Interestingly, the nearby *RAB11FIP2* has been proved to facilitate melanin exocytosis from melanocytes and filopodia-mediated melanin transfer (Beaumont et al., 2011; Tarafder et al., 2014), and SNP rs35563099 192 kb upstream of *RAB11FIP2* also reached a genome-wide significance. To be noted, rs77733715 that has been associated with ease of tanning and darker skin color in UK BioBank samples (Sturm and Duffy, 2018) reached genome-wide significance in the discovery set but failed validation in our cohort. rs77733715 lies near the pigmentary gene *MFSD12* (Crawford et al., 2017; Adhikari et al., 2019; Hédan et al., 2019; Tanaka et al., 2019) and is in LD with the well-documented pigmentation-associated missense variant *MFSD12* rs2240751 (*r* ^2^ = 0.56) in the Korean and Latin American populations (Adhikari et al., 2019; Shin et al., 2021).

The study also has room for improvement. First, the individuals were recruited based on their self-reported traits instead of professional assessment. As the traits included are easily distinguished aesthetic traits and illustrations were added for each question, we consider the reports reliable. Second, functional annotation suggested that some of the variants may be associated with phenotype by impacting the expression level or coding sequence of the nearby genes, but the functions of these variants should be further determined and experimentally evaluated. Moreover, SNP heritability was presented for each trait, but since it can only include contributions from causal variants tagged by the measured SNPs, it is lower than total narrow-sense heritability, such as estimation from twin or family studies. For instance, an adult twin study on the relative contribution of genetic and environmental effects on the expression of nevi and freckles suggested that additive genetic effects explained 91% of the variance in freckle counts (Bataille et al., 2000). Future studies on narrow-sense heritability could be adopted to understand the genetic contribution of the five aesthetic facial traits.

## 5 Conclusion

This GWAS of five aesthetic facial traits in a large Chinese cohort of 26,806 uncovered ten novel genetic associations. Specifically, this is the first study, to our knowledge, to report genetic predispositions to widow’s peak. The identified variants indicated both important similarities and differences among different ethnic groups. Hopefully, the findings would facilitate an understanding of the genetic basis of facial traits and, more importantly, facial development.

## Data Availability

The GWAS summary statistics can be found at the publicly available at http://www.biosino.org/node/project/detail/OEP002975.
