# Supplementary material for: Novel genetic associations with five aesthetic facial traits: A genome-wide association study in the Chinese population
Source: Front Genet. 2022 Aug 12;13:967684. doi: 10.3389/fgene.2022.967684 (PMC9411802; doi:10.3389/fgene.2022.967684)
Supplement: Supplementary file 1 [file DataSheet1.PDF]

# Supplementary Material

## SUPPLEMENTARY METHODS

### Subject, sample, and phenotypes

Volunteers aged 18 to 70 years were enrolled and grouped according to their answers to the questionnaires. Some phenotypes were queried as “Please select your state of the constitution (with respect to certain trait).” Traits queried like this included freckles (Chinese: Que Ban) and widow’s peak (Chinese: Mei Ren Jian), and the choices were set as “applicable”, “not applicable”, and “not sure”. Individuals answering “applicable” were considered cases and those answering “not applicable” as controls, while those answering “not sure” were excluded from the present research. The other set of questions were set in the form “Please select the condition you are closest to.” The traits included earlobe attachment (Chinese: Lian Er Chui), unibrow (Chinese: Yi Zi Mei), and double eyelid (Chinese: Shuang Yan Pi). For earlobe attachment, the options were as follows: A. Free earlobe that hangs below the point of ear attachment to the head; B. Attached earlobe that blends in with the side of the head; C. Not sure. For unibrow, the options were: A. Separated eyebrows; B. Severe unibrow; C. Moderate unibrow; D. Mild unibrow; E. Not sure. For each of these phenotypes, individuals selecting “A” were considered as controls, those answering “Not sure” were excluded and those selecting the other options were included as cases. For double eyelid, the options were as follows: A. Outfold double eyelid with crease connected to the inner canthus; B. Outfold double eyelid with crease not connected to the inner canthus; C. Demi-double eyelid; D. Single eyelid; E. Not sure. Individuals selecting “A” or “B” were set as controls, those selecting “C” were cases, and those choosing “D” or “E” were excluded. Specifically, an extra question was asked for quality control: “Do you have the same type of eyelid now as when you were a kid?”, and individuals answering “No” were excluded. All the questions were set as single-choice questions, and each option was followed by a picture illustrating the manifestation of the corresponding phenotype.

Additional personal information such as sex and age of the participants was also collected in the survey for potential use as covariates and participants’ filtering conditions in data analysis.

### Ethics

Informed consents were electronically signed by all participants to allow the volunteers’ survey answers and genotypic information to be used for research purposes only. The study strictly abided by the Regulation of the People's Republic of China on the Administration of Human Genetic Resources, and was approved by the local ethics committee (West China Hospital, Sichuan University, approval no. 2017(241)). Individual data privacy was strictly protected, and all genotyping and data analysis were completed in China. All acts were performed in compliance with the relevant guidelines and regulations.

## Genotyping and quality control

DNA samples passing quality control (QC) according to the OD260/OD280 ratio by spectrophotometry and the integrity in agarose gels were submitted for genotyping using three highly Affymetrix Axiom Precision Medicine Research Array (PMRA)-based high-throughput gene chips (Chip v1.0, Chipv2.0, and Chip v2.1) (Affymetrix, Santa Clara, CA, U.S.A.) using a GeneTitan Multi-Channel (MC) Instrument (Thermo Fisher Scientific). The gene chips cover about 800,000 SNPs and Insert and Deletions (InDels) spanning 22 pairs of autosomes, 2 sex chromosomes, and the mitochondrial DNA.

To control the genotyping quality at the individual level, data from participants with a missing genotype call rate (CR) $>$ 2% were excluded. Individuals with gender inconsistencies (different between Plink imputed and individually reported) were also filtered out. Meanwhile, individuals who deviated more than ±3 SD from the samples' heterozygosity rate mean and those with cryptic relatedness were also considered failed and excluded. Outliers identified by the multidimensional scaling (MDS) approach were also removed (Supplementary Figure 1).

Subsequently, for a single SNP, the raw dataset was subjected to a series of quality checks. We kept autosomal SNPs only and then performed data analysis to filter out SNPs with CR $<$ 0.98 and minor allele frequency (MAF) $<$ 0.01. A test of the Hardy-Weinberg equilibrium (HWE) was also applied to exclude potential substructure or genotyping errors (HWE control threshold was $1 \times 10^{-6}$).

Missing values were then imputed with Minimac4 v1.0.0 on the basis of the reference genotypes of the 1000 Genomes Project (Genomes Project et al., 2015; Das et al., 2016) and SNPs with $r^2 < 0.7$ or MAF $< 0.01$ were filtered out (Supplementary Figure 1).

## GWAS analysis and functional annotation

In the genome-wide association study phase, 80% of the samples were used for discovering variants associated with the five facial traits (widow's peak, unibrow, double eyelid, earlobe association, and freckles), together with sex, age, and five top principal components (PCs) as covariates, and the other 20% were used for validation. Additional QC was further performed before the association analyses, including the inclusion of SNPs with CR ≥ 0.98 and MAF ≥ 0.01, removal of heterozygosity outliers, removal of individuals with cryptic relatedness, and population structure outliers. The genotype frequency between cases and controls was compared by the logistic regression model, using PLINK v1.90b5.4. Two-sided *P*-values, per-allele odds ratios (ORs), and a 95% confidence interval (CI) were generated. *P*-values $< 5 \times 10^{-8}$ were considered as genome-wide significant and the corresponding candidate SNPs were put into tables and subjected to the construction of Manhattan plots. The prevalence of each phenotype according to ventile groups (e.g. bottom 5%) of the testing test were plotted as well.

To predict the functional potential, the genome-wide SNPs were subjected to HaploReg database (https://pubs.broadinstitute.org/mammals/haploreg/haploreg.php) (Ward and Kellis, 2012). The WashU EpiGenome Browser (http://epigenomegateway.wustl.edu/browser/) (Zhou et al., 2015) were used to annotate the epigenetic landscape, and multi-tissue expression quantitative trait loci (eQTL) Metasoft results were downloaded from the Genotype-Tissue Expression (GTEx) dataset (https://www.gtexportal.org/home/datasets) to assess the eQTL associations ( Consortium, G., 2015).

## Genome-wide polygenic score analysis

To derive the polygenic risk scores, the GWAS discovery set with 80% samples and the discovery GWAS statistics were used. For each of the five traits, in the range of seven *P*-values ($1\times10^{-2}$, $1\times10^{-3}$, $1\times10^{-4}$, $1\times10^{-5}$, $1\times10^{-6}$, $1\times10^{-7}$, and $1\times10^{-8}$) and four $r^2$ thresholds in the linkage disequilibrium (LD) reference provided by an independent cohort with 5000 samples of Chinese ancestry (0.2, 0.4, 0.6, and 0.8), we derived 28 (4×7) GPS predictors based on the pruning and thresholding (P-T) approach (Vilhjálmsson et al., 2015). The scores were acquired by multiplying the genotype dose of each risk allele at each locus by its respective association estimate betas (log-odds) as weights and summing up at all loci. For each trait, area under curve (AUC) of each predictor was determined and the best predictor with the maximal AUC in the discovery set was determined. Afterward, the best GPS predictors were applied to the validation set containing the other 20% samples to calculate the corrected polygenic scores and assess the performance of the GPSs. The GPSs were determined using a logistic regression model adjusted for age, sex, and five top PCs. The individuals in the validation set were binned into 20 groups according to the quantile of the GPS distribution and the prevalence of each trait within each quantile was calculated as the average probability of all individuals within that bin (Khera et al., 2018). Odds ratios (ORs) based on the quantile were calculated as the ratios of highest to the average GPS, the lowest to the average GPS, and the highest to the lowest GPS, respectively.

Supplementary material is not typeset so please ensure that all information is clearly presented, the appropriate caption is included in the file and not in the manuscript, and that the style conforms to the rest of the article. To avoid discrepancies between the published article and the supplementary material, please do not add the title, author list, affiliations or correspondence in the supplementary files.

## References


Consortium, G. (2015). Human genomics. The Genotype-Tissue Expression (GTEx) pilot analysis: multitissue gene regulation in humans. *Science* 348(6235)**,** 648-660. doi: 10.1126/science.1262110.

Das, S., Forer, L., Schonherr, S., Sidore, C., Locke, A.E., Kwong, A., et al. (2016). Next-generation genotype imputation service and methods. *Nat Genet* 48(10)**,** 1284-1287. doi: 10.1038/ng.3656.

Genomes Project, C., Auton, A., Brooks, L.D., Durbin, R.M., Garrison, E.P., Kang, H.M., et al. (2015). A global reference for human genetic variation. *Nature* 526(7571)**,** 68-74. doi: 10.1038/nature15393.

Khera, A.V., Chaffin, M., Aragam, K.G., Haas, M.E., Roselli, C., Choi, S.H., et al. (2018). Genome-wide polygenic scores for common diseases identify individuals with risk equivalent to monogenic mutations. *Nature Genetics* 50(9)**,** 1219-1224. doi: 10.1038/s41588-018-0183-z.

Vilhjálmsson, B.J., Yang, J., Finucane, H.K., Gusev, A., Lindström, S., Ripke, S., et al. (2015). Modeling Linkage Disequilibrium Increases Accuracy of Polygenic Risk Scores. *Am J Hum Genet* 97(4)**,** 576-592. doi: 10.1016/j.ajhg.2015.09.001.

Ward, L.D., and Kellis, M. (2012). HaploReg: a resource for exploring chromatin states, conservation, and regulatory motif alterations within sets of genetically linked variants. *Nucleic Acids Res* 40(Database issue)**,** D930-934. doi: 10.1093/nar/gkr917.

Zhou, X., Li, D., Zhang, B., Lowdon, R.F., Rockweiler, N.B., Sears, R.L., et al. (2015). Epigenomic annotation of genetic variants using the Roadmap Epigenome Browser. *Nat Biotechnol* 33(4), 345-346. doi: 10.1038/nbt.3158.

**Supplementary Table 1.** Minor allele frequency (MAF) of the candidate SNPs.

| rs ID | MAF (%) | | | | | | | | | | |
|---|---|---|---|---|---|---|---|---|---|---|---|
| | Global | European | East Asian | African | American | Test (Case) | Test (Control) | Test | Train (Case) | Train (Control) | Train |
| **Widow's Peak** | | | | | | | | | | | |
| rs13423753 | 0.16 | 0.05 | 0.28 | 0.22 | 0.07 | 0.25 | 0.30 | 0.27 | 0.24 | 0.29 | 0.26 |
| rs4662351 | 0.33 | 0.47 | 0.31 | 0.08 | 0.35 | 0.33 | 0.34 | 0.34 | 0.31 | 0.35 | 0.33 |
| rs4959669 | 0.11 | 0.13 | 0.12 | 0.07 | 0.08 | 0.09 | 0.17 | 0.13 | 0.08 | 0.15 | 0.11 |
| rs78539508 | 0.06 | 0.01 | 0.18 | 0.00 | 0.00 | 0.15 | 0.15 | 0.15 | 0.14 | 0.17 | 0.15 |
| rs12618491 | 0.46 | 0.35 | 0.64 | 0.28 | 0.47 | 0.44 | 0.40 | 0.42 | 0.44 | 0.40 | 0.42 |
| rs6869535 | 0.18 | 0.16 | 0.08 | 0.26 | 0.19 | 0.05 | 0.06 | 0.05 | 0.04 | 0.06 | 0.05 |
| rs7128448 | 0.46 | 0.40 | 0.69 | 0.36 | 0.39 | 0.33 | 0.32 | 0.33 | 0.35 | 0.31 | 0.33 |
| rs71118502 | 0.19 | 0.40 | 0.11 | 0.06 | 0.23 | 0.13 | 0.11 | 0.12 | 0.13 | 0.10 | 0.11 |
| **Unibrow** | | | | | | | | | | | |
| rs36015125 | 0.22 | 0.13 | 0.55 | 0.01 | 0.26 | 0.49 | 0.43 | 0.46 | 0.48 | 0.43 | 0.47 |
| rs10205370 | 0.51 | 0.43 | 0.95 | 0.15 | 0.62 | 0.03 | 0.04 | 0.03 | 0.03 | 0.05 | 0.04 |
| rs10592167 | 0.11 | 0.03 | 0.18 | 0.13 | 0.09 | 0.19 | 0.19 | 0.19 | 0.16 | 0.20 | 0.18 |
| rs4959668 | 0.11 | 0.13 | 0.12 | 0.09 | 0.08 | 0.11 | 0.13 | 0.12 | 0.11 | 0.14 | 0.13 |
| rs12550594 | 0.34 | 0.20 | 0.73 | 0.24 | 0.41 | 0.28 | 0.26 | 0.27 | 0.27 | 0.23 | 0.25 |
| rs57721278 | 0.12 | 0.04 | 0.41 | 0.00 | 0.15 | 0.38 | 0.40 | 0.39 | 0.36 | 0.41 | 0.39 |
| rs61930443 | 0.12 | 0.20 | 0.07 | 0.08 | 0.11 | 0.06 | 0.07 | 0.06 | 0.05 | 0.07 | 0.06 |
| rs62113423 | 0.13 | 0.17 | 0.03 | 0.19 | 0.25 | 0.03 | 0.02 | 0.03 | 0.04 | 0.03 | 0.03 |
| **Double Eyelid** | | | | | | | | | | | |
| rs7549180 | 0.16 | 0.02 | 0.14 | 0.46 | 0.05 | 0.15 | 0.12 | 0.13 | 0.16 | 0.11 | 0.13 |
| rs10749244 | 0.42 | 0.61 | 0.51 | 0.18 | 0.50 | 0.41 | 0.48 | 0.45 | 0.41 | 0.48 | 0.46 |
| rs79852633 | 0.04 | 0.01 | 0.13 | 0.00 | 0.02 | 0.16 | 0.11 | 0.12 | 0.15 | 0.11 | 0.13 |
| rs6499632 | 0.65 | 0.49 | 0.63 | 0.93 | 0.54 | 0.45 | 0.39 | 0.41 | 0.44 | 0.37 | 0.40 |
| rs147581439 | 0.00 | 0.00 | 0.03 | 0.00 | 0.00 | 0.03 | 0.02 | 0.02 | 0.03 | 0.02 | 0.02 |
| rs10779357 | 0.72 | 0.67 | 0.76 | 0.80 | 0.50 | 0.30 | 0.28 | 0.29 | 0.32 | 0.27 | 0.29 |
| rs137959381 | 0.00 | 0.00 | 0.01 | 0.00 | 0.00 | 0.02 | 0.01 | 0.01 | 0.01 | 0.02 | 0.02 |
| rs10030259 | 0.55 | 0.60 | 0.53 | 0.47 | 0.62 | 0.48 | 0.50 | 0.49 | 0.48 | 0.47 | 0.49 |

| | | | | | | | | | | | |
|---|---|---|---|---|---|---|---|---|---|---|---|
| rs10875601 | 0.70 | 0.92 | 0.72 | 0.39 | 0.89 | 0.31 | 0.28 | 0.29 | 0.30 | 0.25 | 0.27 |
| **Earlobe Attachment** | | | | | | | | | | | |
| rs3827760 | 0.24 | 0.01 | 0.87 | 0.00 | 0.39 | 0.06 | 0.08 | 0.07 | 0.06 | 0.09 | 0.08 |
| rs10198822 | 0.42 | 0.60 | 0.60 | 0.15 | 0.37 | 0.47 | 0.36 | 0.41 | 0.46 | 0.37 | 0.41 |
| rs10211400 | 0.18 | 0.04 | 0.14 | 0.42 | 0,050 | 0.14 | 0.17 | 0.16 | 0.13 | 0.17 | 0.15 |
| rs12695694 | 0.30 | 0.23 | 0.45 | 0.04 | 0.50 | 0.39 | 0.48 | 0.44 | 0.39 | 0.49 | 0.45 |
| rs9496426 | 0.26 | 0.26 | 0.24 | 0.37 | 0.24 | 0.24 | 0.18 | 0.21 | 0.25 | 0.19 | 0.22 |
| rs17105172 | 0.17 | 0.10 | 0.26 | 0.05 | 0.18 | 0.26 | 0.23 | 0.25 | 0.27 | 0.23 | 0.25 |
| rs74030209 | 0.11 | 0.01 | 0.33 | 0.07 | 0.01 | 0.31 | 0.35 | 0.33 | 0.29 | 0.34 | 0.32 |
| rs2742261 | 0.75 | 0.64 | 0.63 | 0.97 | 0.69 | 0.33 | 0.38 | 0.36 | 0.32 | 0.37 | 0.35 |
| rs191057905 | 0.03 | 0.00 | 0.10 | 0.00 | 0.04 | 0.07 | 0.07 | 0.07 | 0.09 | 0.06 | 0.07 |
| rs12700650 | 0.77 | 0.68 | 0.85 | 0.77 | 0.80 | 0.17 | 0.14 | 0.16 | 0.18 | 0.15 | 0.16 |
| rs6476623 | 0.44 | 0.37 | 0.28 | 0.69 | 0.31 | 0.28 | 0.29 | 0.29 | 0.31 | 0.27 | 0.29 |
| **Freckles** | | | | | | | | | | | |
| rs251468 | 0.33 | 0.26 | 0.22 | 0.42 | 0.41 | 0.16 | 0.23 | 0.21 | 0.16 | 0.22 | 0.21 |
| rs4455968 | 0.18 | 0.08 | 0.32 | 0.06 | 0.31 | 0.37 | 0.30 | 0.32 | 0.36 | 0.30 | 0.31 |
| rs12245621 | 0.25 | 0.22 | 0.22 | 0.20 | 0.24 | 0.25 | 0.20 | 0.21 | 0.27 | 0.20 | 0.22 |
| rs35563099 | 0.15 | 0.15 | 0.10 | 0.19 | 0.10 | 0.06 | 0.11 | 0.10 | 0.06 | 0.09 | 0.08 |
| rs112854838 | 0.02 | 0.01 | 0.05 | 0.00 | 0.01 | 0.07 | 0.06 | 0.06 | 0.07 | 0.05 | 0.05 |
| rs35415928 | 0.08 | 0.08 | 0.11 | 0.01 | 0.13 | 0.15 | 0.11 | 0.12 | 0.14 | 0.10 | 0.11 |
| rs77733715 | 0.10 | 0.01 | 0.36 | 0.00 | 0.18 | 0.36 | 0.40 | 0.39 | 0.35 | 0.41 | 0.39 |
| rs58944021 | 0.13 | 0.19 | 0.05 | 0.04 | 0.14 | 0.06 | 0.06 | 0.06 | 0.07 | 0.05 | 0.06 |
| rs141675941 | 0.11 | 0.09 | 0.30 | 0.01 | 0.14 | 0.31 | 0.34 | 0.33 | 0.30 | 0.35 | 0.33 |
| rs67000335 | 0.24 | 0.38 | 0.15 | 0.15 | 0.37 | 0.11 | 0.13 | 0.12 | 0.14 | 0.11 | 0.12 |

SNP, single nucleotide polymorphism; MAF, minor allele frequency.

**Supplementary Table 2.** Sample sizes of examined facial traits.

| **Trait** | **Discovery Stage** | | | **Validation Stage** | | | **All** |
|---|---|---|---|---|---|---|---|
| | **Case** | **Control** | **All** | **Case** | **Control** | **All** | |
| **Widow's Peak** | 4,903 | 4,612 | 9,515 | 1,259 | 1,172 | 2,431 | 11,946 |
| **Unibrow** | 2,364 | 3,416 | 5,780 | 603 | 871 | 1,474 | 7,254 |
| **Double Eyelid** | 2,182 | 3,773 | 5,955 | 560 | 958 | 1,518 | 7,473 |
| **Earlobe Attachment** | 3,477 | 4,479 | 7,956 | 890 | 1,131 | 2,021 | 9,977 |
| **Freckles** | 1,548 | 5,031 | 6,579 | 392 | 1,280 | 1,672 | 8,251 |

**Supplementary Table 3.** Association analyses of candidate variants (discovery *P*-value < $1\times10^{-7}$) in the discovery cohort and replication cohort.

| rs ID | Chr | Position[a] | Alleles | OR [95% CI] (Discovery) | *P*-value (Discovery) | OR (Validation) | *P*-value (Validation) | Variant Function | Related Gene(s) | Novelty[b] |
|---|---|---|---|---|---|---|---|---|---|---|
| **Widow's Peak** | | | | | | | | | | |
| rs13423753 | 2 | 66008141 | G/A | 0.78 [0.73-0.83] | $2.99\times10^{-14}$ | 0.80 | $5.65\times10^{-4}$ | intergenic | *SPRED2*, *MIR4778* | unreported |
| rs4662351 | 2 | 144559694 | G/T | 0.84 [0.79-0.89] | $1.42\times10^{-8}$ | 0.97 | $6.20\times10^{-1}$ | intergenic | *ARHGAP15*, *LOC101928386* | unreported |
| rs4959669 | 6 | 2453470 | T/C | 0.49 [0.44-0.54] | $1.29\times10^{-49}$ | 0.50 | $3.87\times10^{-14}$ | intergenic | *GMDS-AS1*, *LINC01600* | unreported |
| rs7128448 | 11 | 69676386 | G/A | 0.85 [0.80-0.91] | $2.93\times10^{-7}$ | 0.96 | $5.22\times10^{-1}$ | intergenic | *FGF3*, *LOC101928443* | unreported |
| rs12618491 | 2 | 223133508 | C/A | 0.86 [0.81-0.91] | $4.59\times10^{-7}$ | 0.84 | $3.04\times10^{-3}$ | intronic | *PAX3* | unreported |
| rs6869535 | 5 | 40597618 | G/A | 0.72 [0.63-0.82] | $7.00\times10^{-7}$ | 0.83 | $1.50\times10^{-1}$ | intergenic | *LINC00603*, *PTGER4* | unreported |
| rs71118502 | 13 | 100403085 | C/CT | 1.26 [1.15-1.38] | $7.05\times10^{-7}$ | 1.25 | $1.57\times10^{-2}$ | intronic | *LOC101927437* | unreported |
| rs78539508 | 2 | 174685316 | T/C | 0.82 [0.75-0.89] | $8.75\times10^{-7}$ | 1.01 | $9.27\times10^{-1}$ | intergenic | *CDCA7*, *SP3* | unreported |
| **Unibrow** | | | | | | | | | | |
| rs36015125 | 2 | 223471268 | C/G | 0.69 [0.63-0.74] | $1.96\times10^{-21}$ | 0.73 | $6.76\times10^{-5}$ | intronic | *FARSB* | unreported |
| rs10592167 | 2 | 174615799 | TGA/T | 0.77 [0.69-0.85] | $2.91\times10^{-7}$ | 0.98 | $8.19\times10^{-1}$ | intergenic | *CDCA7*, *SP3* | unreported |
| rs4959668 | 6 | 2449614 | C/T | 0.74 [0.66-0.83] | $5.49\times10^{-7}$ | 0.76 | $2.80\times10^{-2}$ | intergenic | *GMDS-AS1*, *LINC01600* | unreported |
| rs57721278 | 9 | 129334215 | T/C | 0.82 [0.76-0.89] | $6.07\times10^{-7}$ | 0.87 | $8.05\times10^{-2}$ | intergenic | *MVB12B*, *LMX1B* | unreported |
| rs12550594 | 8 | 129861863 | C/A | 0.80 [0.73-0.87] | $6.14\times10^{-7}$ | 0.88 | $1.19\times10^{-1}$ | intergenic | *LINC00824*, *LINC00976* | unreported |
| rs10205370 | 2 | 109193507 | A/G | 1.74 [1.40-2.17] | $6.74\times10^{-7}$ | 1.36 | $1.64\times10^{-1}$ | intronic | *LIMS1* | unreported |
| rs62113423 | 19 | 7679357 | T/C | 1.71 [1.38-2.11] | $6.91\times10^{-7}$ | 1.50 | $9.37\times10^{-2}$ | intronic | *CAMSAP3* | unreported |
| rs61930443 | 12 | 71379083 | C/T | 0.65 [0.55-0.77] | $9.11\times10^{-7}$ | 0.71 | $3.47\times10^{-2}$ | intergenic | *PTPRR*, *TSPAN8* | unreported |
| **Double Eyelid** | | | | | | | | | | |

| | | | | | | | | | | |
|---|---|---|---|---|---|---|---|---|---|---|
| rs7549180 | 1 | 245376084 | C/A | 1.59 [1.42-1.78] | $2.41\times10^{-15}$ | 1.31 | $2.12\times10^{-2}$ | intronic | *KIF26B* | unreported |
| rs10749244 | 10 | 119344435 | T/C | 1.34 [1.24-1.45] | $1.96\times10^{-13}$ | 1.36 | $1.34\times10^{-4}$ | intergenic | *EMX2*, *RAB11FIP2* | in LD with rs1415425 |
| rs79852633 | 10 | 119837083 | G/A | 1.35 [1.30-1.63] | $4.78\times10^{-11}$ | 1.59 | $5.82\times10^{-5}$ | intronic | *CASC2* | unreported |
| rs6499632 | 16 | 53633081 | T/C | 0.77 [0.71-0.83] | $9.15\times10^{-11}$ | 0.80 | $5.16\times10^{-3}$ | downstream | *RPGRIP1L* | unreported |
| rs147581439 | 20 | 21818113 | G/C | 2.06 [1.59-2.66] | $3.07\times10^{-8}$ | 2.00 | $6.81\times10^{-3}$ | intergenic | *PAX1*, *LINC01432* | unreported |
| rs10779357 | 1 | 219712930 | A/G | 0.79 [0.73-0.86] | $6.89\times10^{-8}$ | 0.94 | $4.77\times10^{-1}$ | intronic | *LOC102723886* | unreported |
| rs10030259 | 4 | 155205511 | C/A | 0.82 [0.76-0.88] | $3.95\times10^{-7}$ | 1.09 | $2.76\times10^{-1}$ | intronic | *DCHS2* | unreported |
| rs137959381 | 2 | 143257876 | T/G | 0.43 [0.30-0.60] | $9.34\times10^{-7}$ | 1.71 | $1.40\times10^{-1}$ | intergenic | *LRP1B*, *KYNU* | unreported |
| rs10875601 | 5 | 142489301 | G/A | 0.80 [0.74-0.88] | $9.58\times10^{-7}$ | 0.85 | $4.87\times10^{-2}$ | intronic | *ARHGAP26* | unreported |
| **Earlobe Attachment** | | | | | | | | | | |
| rs12695694 | 3 | 139000458 | C/G | 0.65 [0.61-0.69] | $5.75\times10^{-39}$ | 0.69 | $1.41\times10^{-8}$ | intergenic | *PISRT1*, *MRPS22* | previously reported |
| rs10198822 | 2 | 171536325 | C/T | 0.68 [0.64-0.73] | $1.70\times10^{-30}$ | 0.62 | $2.09\times10^{-12}$ | intergenic | *MYO3B, LOC101926913* | previously reported |
| rs9496426 | 6 | 142955630 | T/G | 1.40 [1.29-1.51] | $2.09\times10^{-17}$ | 1.44 | $5.27\times10^{-6}$ | intronic | *LOC153910* | in LD with rs28514730 |
| rs74030209 | 16 | 73058926 | C/T | 0.77 [0.72-0.82] | $9.77\times10^{-14}$ | 0.85 | $1.83\times10^{-2}$ | intronic | *ZFHX3* | unreported |
| rs3827760 | 2 | 109513601 | A/G | 1.52 [1.34-1.73] | $4.11\times10^{-11}$ | 1.46 | $3.41\times10^{-3}$ | exonic | *EDAR* | previously reported |
| rs2742261 | 18 | 55230630 | G/A | 1.25 [1.17-1.34] | $6.27\times10^{-11}$ | 1.26 | $9.52\times10^{-4}$ | intronic | *FECH* | in LD with rs1790607 |
| rs10211400 | 2 | 239442903 | G/T | 0.75 [0.69-0.82] | $6.25\times10^{-10}$ | 0.73 | $5.75\times10^{-4}$ | intronic | *LINC01107* | unreported |
| rs17105172 | 14 | 37206476 | G/C | 1.23 [1.15-1.33] | $2.51\times10^{-8}$ | 1.19 | $1.96\times10^{-2}$ | intronic | *SLC25A21* | in LD with rs1950357 |
| rs6476623 | 9 | 37375849 | A/G | 1.21 [1.13-1.29] | $1.45\times10^{-7}$ | 0.95 | $5.07\times10^{-1}$ | intergenic | *ZCCHC7*, *GRHPR* | unreported |
| rs191057905 | 4 | 151198824 | T/A | 1.39 [1.23-1.57] | $1.52\times10^{-7}$ | 0.96 | $7.56\times10^{-1}$ | intronic | LRBA | unreported |
| rs12700650 | 7 | 25655407 | C/T | 0.81 [0.74-0.88] | $8.88\times10^{-7}$ | 0.78 | $5.29\times10^{-3}$ | intergenic | *NPVF*, *MIR148A* | in LD with rs76958973 |
| **Freckles** | | | | | | | | | | |
| rs12245621 | 10 | 118463743 | T/C | 1.46 [1.33-1.61] | $1.64\times10^{-14}$ | 1.33 | $4.80\times10^{-3}$ | intronic | *HSPA12A* | in LD with rs12259842 |
| rs251468 | 5 | 149194485 | C/T | 0.66 [0.59-0.74] | $1.26\times10^{-13}$ | 0.60 | $6.29\times10^{-6}$ | intronic | *PPARGC1B* | previously reported |
| rs4455968 | 9 | 16801450 | G/T | 1.32 [1.21-1.44] | $5.72\times10^{-10}$ | 1.36 | $5.41\times10^{-4}$ | intronic | *BNC2* | in LD with rs10810635 |
| rs77733715 | 19 | 3537184 | A/G | 0.76 [0.70-0.83] | $9.37\times10^{-10}$ | 0.85 | $7.01\times10^{-2}$ | intergenic | *FZR1*, *C19orf71* | previously reported |
| rs35563099 | 10 | 119572403 | C/T | 0.60 [0.51-0.71] | $3.51\times10^{-9}$ | 0.58 | $1.07\times10^{-3}$ | intergenic | *EMX2*, *RAB11FIP2* | previously reported |
| rs112854838 | 16 | 89663715 | G/A | 1.65 [1.39-1.97] | $1.05\times10^{-8}$ | 1.14 | $4.45\times10^{-1}$ | downstream | *CPNE7* | unreported |
| rs35415928 | 16 | 89724268 | C/T | 1.43 [1.26-1.61] | $1.08\times10^{-8}$ | 1.34 | $1.57\times10^{-2}$ | intronic | *SPATA33* | unreported |
| rs67000335 | 8 | 132350424 | A/T | 1.40 [1.24-1.58] | $1.07\times10^{-7}$ | 0.83 | $1.48\times10^{-1}$ | intergenic | *SASH1* | unreported |
| rs141675941 | 6 | 148681034 | A/G | 0.80 [0.73-0.87] | $6.80\times10^{-7}$ | 0.82 | $2.86\times10^{-2}$ | intronic | *SASH1* | unreported |

| | | | | | | | | | | |
|---|---|---|---|---|---|---|---|---|---|---|
| rs58944021 | 3 | 121683518 | ACT/A | 1.52 [1.28-1.79] | $9.18\times10^{-7}$ | 1.07 | $7.34\times10^{-1}$ | intergenic | *SLC15A2*, *ILDR1* | unreported |

Variants that passed validation (validation *P*-value < 0.05) are underlined.

[a]Genomic positions are given according to the human reference genome hg19/GRCh37. [b]single nucleotide polymorphisms (SNPs) in LD with the reported SNPs are recorded. Chr, Chromosome; OR, odds ratio; 95% CI, 95% confidence interval of the odds ratio; LD, linkage disequilibrium.

**Supplementary Table 4.** Coding variants in linkage disequilibrium (LD) with genome-wide significant variants.

| rs ID | Variant in LD[a] | Chr | Position[b] | LD ($r^2$) | Ref | Alt | MAF (Asian) | Motifs changed | GENCODE gene | dbSNP function annotation |
|---|---|---|---|---|---|---|---|---|---|---|
| **Double Eyelid** | | | | | | | | | | |
| rs6499632 | rs3213758 | 16 | 53639438 | 0.26 | C | T | 0.27 | ERalpha-a, THAP1, YY1 | *RPGRIP1L* | missense |
| **Earlobe Attachment** | | | | | | | | | | |
| rs3827760 | rs6740879 | 2 | 109408208 | 0.22 | G | A | 0.09 | CDP, Homez | *CCDC138* | missense |
| | rs146567337 | 2 | 109513572 | 0.21 | T | G | 0.03 | 4 altered motifs | *EDAR* | missense |
| | rs3827760 | 2 | 109513601 | 1.00 | A | G | 0.87 | CEBPA, Nanog | *EDAR* | missense |
| rs10211400 | rs3769110 | 2 | 239237751 | 0.28 | A | G | 0.11 | Smad3, Smad | *TRAF3IP1* | missense |
| | rs17854985 | 2 | 239237974 | 0.28 | T | C | 0.11 | E2F | *TRAF3IP1* | synonymous |
| | rs58277463 | 2 | 239253224 | 0.28 | A | T | 0.11 | CCNT2 | *TRAF3IP1* | missense |
| rs74030209 | rs10852515 | 16 | 72991660 | 0.26 | C | G | 0.68 | 6 altered motifs | *ZFHX3* | synonymous |
| | rs4788682 | 16 | 72991715 | 0.23 | A | G | 0.66 | 15 altered motifs | *ZFHX3* | missense |
| | rs7193297 | 16 | 72993831 | 0.25 | A | C | 0.26 | 12 altered motifs | *ZFHX3* | missense |
| rs2742261 | rs536560 | 18 | 55221648 | 0.75 | T | C | 0.67 | | *FECH* | synonymous |
| | rs536765 | 18 | 55226383 | 0.89 | G | C | 0.64 | PU.1, TCF11::MafG | *FECH* | synonymous |
| **Freckles** | | | | | | | | | | |
| rs251468 | rs32588 | 5 | 149200043 | 0.29 | T | C | 0.08 | 4 altered motifs | *PPARGC1B* | synonymous |
| | rs45588534 | 5 | 149216256 | 0.55 | C | T | 0.14 | E2F, TCF12, ZID | *PPARGC1B* | synonymous |
| | rs45543631 | 5 | 149216304 | 0.55 | C | T | 0.14 | CAC-binding-protein, Hic1 | *PPARGC1B* | synonymous |
| rs4455968 | rs10962592 | 9 | 16787039 | 0.28 | G | A | 0.17 | 10 altered motifs | *BNC2* | missense |

[a]Variants with $r^2 \geq 0.2$ are considered to be in LD with the identified SNP. [b]Genomic positions are given according to the human reference genome hg19/GRCh37. Chr, Chromosome; MAF, minor allele frequency; SNP, single nucleotide polymorphism.

**Supplementary Table 5.** Expression quantitative trait locus (eQTL) information and epigenetic landscape of the genome-wide significant variants.

| rs ID | Chr | GTEx eQTL Association: Gene(s) | Cells / Tissue(s) | *P*-value | Epigenetic Information: Epidermal Keratinocyte Primary Cells | Dermal Fibroblast Primary Cells |
|---|---|---|---|---|---|---|
| **Widow's Peak** | | | | | | |
| rs13423753 | 2 | - | | | quiescent | weak repressed polycomb |
| rs4959669 | 6 | - | | | weak repressed polycomb | weak repressed polycomb |
| **Unibrow** | | | | | | |
| rs36015125 | 2 | *RP11-16P6.1* | Skin - Sun Exposed (Lower leg) | $1.60{\times}10^{-19}$ | weak transcription | weak transcription |
| | | | Skin - Not Sun Exposed (Suprapubic) | $5.40{\times}10^{-19}$ | | |
| | | | Cells - Cultured fibroblasts | $4.50{\times}10^{-16}$ | | |
| | | *SGPP2* | Skin - Not Sun Exposed (Suprapubic) | $3.40{\times}10^{-7}$ | | |
| | | | Skin - Sun Exposed (Lower leg) | $1.70{\times}10^{-6}$ | | |
| | | | Cells - Cultured fibroblasts | $1.20{\times}10^{-5}$ | | |
| | | *FARSB* | Cells - Cultured fibroblasts | $1.40{\times}10^{-5}$ | | |
| **Double Eyelid** | | | | | | |
| rs7549180 | 1 | - | | | heterochromatin | quiescent |
| rs10749244 | 10 | - | | | quiescent | quiescent |
| rs79852633 | 10 | - | | | quiescent | quiescent |
| rs6499632 | 16 | *RP11-36I17.2* | Cells - Cultured fibroblasts | $4.30{\times}10^{-5}$ | weak enhancer | weak enhancer |
| rs147581439 | 20 | - | | | heterochromatin | quiescent |
| **Earlobe Attachment** | | | | | | |
| rs3827760 | 2 | - | | | quiescent | weak repressed polycomb |
| rs10198822 | 2 | - | | | quiescent | quiescent |
| rs10211400 | 2 | - | | | heterochromatin | weak repressed polycomb |
| rs12695694 | 3 | *MRPS22* | Cells - Cultured fibroblasts | $7.70{\times}10^{-6}$ | repressed polycomb | active transcription start site |
| rs9496426 | 6 | - | | | quiescent | weak repressed polycomb |
| rs17105172 | 14 | - | | | weak repressed polycomb | weak repressed polycomb |
| rs74030209 | 16 | - | | | quiescent | weak enhancer |
| rs2742261 | 18 | *NARS* | Cells - Cultured fibroblasts | $1.10{\times}10^{-5}$ | weak transcription | weak transcription |
| | | *FECH* | Cells - Cultured fibroblasts | $4.10{\times}10^{-5}$ | | |

| | | | | | | |
|---|---|---|---|---|---|---|
| **Freckles** | | | | | | |
| rs251468 | 5 | - | | | weak transcription | quiescent |
| rs4455968 | 9 | - | | | quiescent | quiescent |
| rs12245621 | 10 | *HSPA12A* | Skin - Sun Exposed (Lower leg) | $2.00\times10^{-25}$ | quiescent | weak transcription |
| | | | Skin - Not Sun Exposed (Suprapubic) | $2.60\times10^{-11}$ | | |
| rs35563099 | 10 | - | | | heterochromatin | quiescent |
| rs35415928 | 16 | *DBNDD1* | Cells - Cultured fibroblasts | $9.20\times10^{-19}$ | active transcription start site | active transcription start site |
| | | | Skin - Sun Exposed (Lower leg) | $5.50\times10^{-14}$ | | |
| | | | Skin - Not Sun Exposed (Suprapubic) | $3.00\times10^{-13}$ | | |
| | | *CDK10* | Skin - Not Sun Exposed (Suprapubic) | $6.90\times10^{-9}$ | | |
| | | *GAS8* | Skin - Sun Exposed (Lower leg) | $1.50\times10^{-8}$ | | |
| | | *FANCA* | Cells - Cultured fibroblasts | $9.50\times10^{-8}$ | | |
| | | | Skin - Not Sun Exposed (Suprapubic) | $9.20\times10^{-5}$ | | |
| | | *CHMP1A* | Skin - Sun Exposed (Lower leg) | $1.60\times10^{-6}$ | | |
| | | | Skin - Not Sun Exposed (Suprapubic) | $8.00\times10^{-5}$ | | |
| | | *RNF166* | Skin - Sun Exposed (Lower leg) | $5.90\times10^{-5}$ | | |
| | | *VPS9D1* | Skin - Sun Exposed (Lower leg) | $5.90\times10^{-5}$ | | |
| | | | Cells - Cultured fibroblasts | $8.40\times10^{-5}$ | | |

Chr, Chromosome; GTEx, Genotype-Tissue Expression; eQTL, expression quantitative trait locus.

**Supplementary Table 6.** Genome-wide polygenic predictors derived from discovery genome-wide association statistics using pruning and thresholding (P-T) methods.

| Tuning Parameter | | AUC (95%CI)[a] | | | | |
|---|---|---|---|---|---|---|
| *P*-value | $r^2$ | Widow's Peak | Unibrow | Double Eyelid | Earlobe Attachment | Freckles |
| 1.00×10-8 | 0.2 | 0.589 (0.566-0.611) | 0.556 (0.527-0.586) | 0.605 (0.576-0.634) | 0.630 (0.606-0.655) | 0.606 (0.574-0.638) |
| 1.00×10-8 | 0.4 | 0.585 (0.563-0.608) | 0.552 (0.523-0.581) | 0.600 (0.571-0.629) | 0.640 (0.616-0.664) | 0.618 (0.586-0.650) |
| 1.00×10-8 | 0.6 | 0.596 (0.574-0.619) | 0.550 (0.521-0.579) | 0.603 (0.574-0.633) | 0.635 (0.611-0.659) | 0.604 (0.573-0.636) |
| 1.00×10-8 | 0.8 | 0.589 (0.566-0.611) | 0.556 (0.527-0.586) | 0.605 (0.576-0.634) | 0.637 (0.613-0.661) | 0.606 (0.574-0.638) |
| 1.00×10-7 | 0.2 | 0.588 (0.566-0.611) | 0.554 (0.525-0.584) | 0.614 (0.585-0.643) | 0.636 (0.611-0.660) | 0.616 (0.584-0.647) |
| **1.00×10-7** | **0.4** | 0.586 (0.563-0.608) | 0.552 (0.523-0.581) | 0.618 (0.589-0.647) | 0.646 (0.622-0.670) | **0.625 (0.593-0.656)** |
| 1.00×10-7 | 0.6 | 0.596 (0.574-0.619) | 0.550 (0.521-0.579) | 0.615 (0.586-0.644) | 0.640 (0.616-0.664) | 0.615 (0.583-0.646) |
| 1.00×10-7 | 0.8 | 0.588 (0.566-0.611) | 0.554 (0.525-0.584) | 0.614 (0.585-0.643) | 0.639 (0.615-0.663) | 0.616 (0.584-0.647) |
| 1.00×10-6 | 0.2 | 0.587 (0.564-0.610) | 0.565 (0.535-0.594) | 0.622 (0.594-0.651) | 0.637 (0.613-0.661) | 0.620 (0.588-0.651) |
| 1.00×10-6 | 0.4 | 0.588 (0.565-0.610) | 0.569 (0.539-0.598) | 0.619 (0.590-0.647) | 0.648 (0.624-0.672) | 0.621 (0.589-0.653) |
| 1.00×10-6 | 0.6 | 0.595 (0.572-0.617) | 0.567 (0.537-0.596) | 0.621 (0.592-0.649) | 0.643 (0.619-0.667) | 0.620 (0.589-0.652) |
| 1.00×10-6 | 0.8 | 0.587 (0.564-0.610) | 0.565 (0.535-0.594) | 0.622 (0.594-0.651) | 0.642 (0.618-0.666) | 0.620 (0.588-0.651) |
| 1.00×10-5 | 0.2 | 0.593 (0.570-0.615) | 0.588 (0.558-0.617) | 0.645 (0.617-0.674) | 0.65 (0.626-0.674) | 0.610 (0.578-0.642) |
| **1.00×10-5** | **0.4** | 0.587 (0.564-0.609) | **0.594 (0.565-0.624)** | 0.638 (0.609-0.666) | **0.657 (0.633-0.681)** | 0.600 (0.568-0.633) |
| **1.00×10-5** | **0.6** | **0.598 (0.576-0.621)** | 0.588 (0.559-0.617) | 0.642 (0.613-0.670) | 0.648 (0.624-0.672) | 0.608 (0.576-0.641) |
| 1.00×10-5 | 0.8 | 0.593 (0.570-0.615) | 0.588 (0.558-0.617) | 0.645 (0.617-0.674) | 0.647 (0.623-0.671) | 0.610 (0.578-0.642) |
| **1.00×10-4** | **0.2** | 0.593 (0.571-0.616) | 0.590 (0.560-0.619) | **0.665 (0.637-0.692)** | 0.636 (0.612-0.660) | 0.617 (0.586-0.649) |
| 1.00×10-4 | 0.4 | 0.591 (0.568-0.613) | 0.580 (0.551-0.61) | 0.643 (0.615-0.671) | 0.647 (0.623-0.671) | 0.605 (0.573-0.637) |
| 1.00×10-4 | 0.6 | 0.595 (0.573-0.618) | 0.586 (0.556-0.615) | 0.650 (0.622-0.678) | 0.649 (0.625-0.673) | 0.619 (0.587-0.651) |
| **1.00×10-4** | **0.8** | 0.593 (0.571-0.616) | 0.590 (0.560-0.619) | **0.665 (0.637-0.692)** | 0.650 (0.626-0.674) | 0.617 (0.586-0.649) |
| 1.00×10-3 | 0.2 | 0.581 (0.559-0.604) | 0.581 (0.551-0.610) | 0.636 (0.607-0.664) | 0.605 (0.581-0.630) | 0.572 (0.538-0.605) |
| 1.00×10-3 | 0.4 | 0.577 (0.555-0.600) | 0.569 (0.539-0.598) | 0.613 (0.584-0.642) | 0.619 (0.595-0.644) | 0.570 (0.537-0.603) |
| 1.00×10-3 | 0.6 | 0.585 (0.562-0.608) | 0.575 (0.545-0.604) | 0.627 (0.598-0.655) | 0.636 (0.612-0.660) | 0.575 (0.542-0.608) |
| 1.00×10-3 | 0.8 | 0.581 (0.559-0.604) | 0.581 (0.551-0.610) | 0.636 (0.607-0.664) | 0.630 (0.606-0.654) | 0.572 (0.538-0.605) |
| 1.00×10-2 | 0.2 | 0.564 (0.541-0.587) | 0.550 (0.521-0.580) | 0.601 (0.572-0.631) | 0.563 (0.538-0.588) | 0.542 (0.509-0.575) |
| 1.00×10-2 | 0.4 | 0.563 (0.540-0.585) | 0.560 (0.531-0.590) | 0.595 (0.566-0.625) | 0.571 (0.546-0.596) | 0.559 (0.527-0.591) |

| | | | | | | |
|---|---|---|---|---|---|---|
| 1.00×10-2 | 0.6 | 0.567 (0.545-0.590) | 0.561 (0.532-0.591) | 0.601 (0.572-0.631) | 0.593 (0.568-0.618) | 0.567 (0.534-0.599) |
| 1.00×10-2 | 0.8 | 0.564 (0.541-0.587) | 0.550 (0.521-0.580) | 0.593 (0.564-0.623) | 0.589 (0.564-0.614) | 0.542 (0.509-0.575) |

[a]The score with the highest AUC for each trait is denoted by the bolded font. AUC, area under curve; 95% CI, 95% confidence interval of the AUC.

**Supplementary Table 7.** Odds ratio (OR) according to quantile of the genome-wide polygenic scores (GPSs).

| Trait | Highest/Average | Lowest/Average | Highest/Lowest |
|---|---|---|---|
| **Widow's Peak** | 1.94 | 0.64 | 3.01 |
| **Unibrow** | 2.00 | 0.46 | 4.32 |
| **Double Eyelid** | 1.21 | 0.44 | 2.74 |
| **Earlobe Attachment** | 1.61 | 0.66 | 2.42 |
| **Freckles** | 1.72 | 0.84 | 2.05 |

OR, odds ratio; GPS, genome-wide polygenic score.

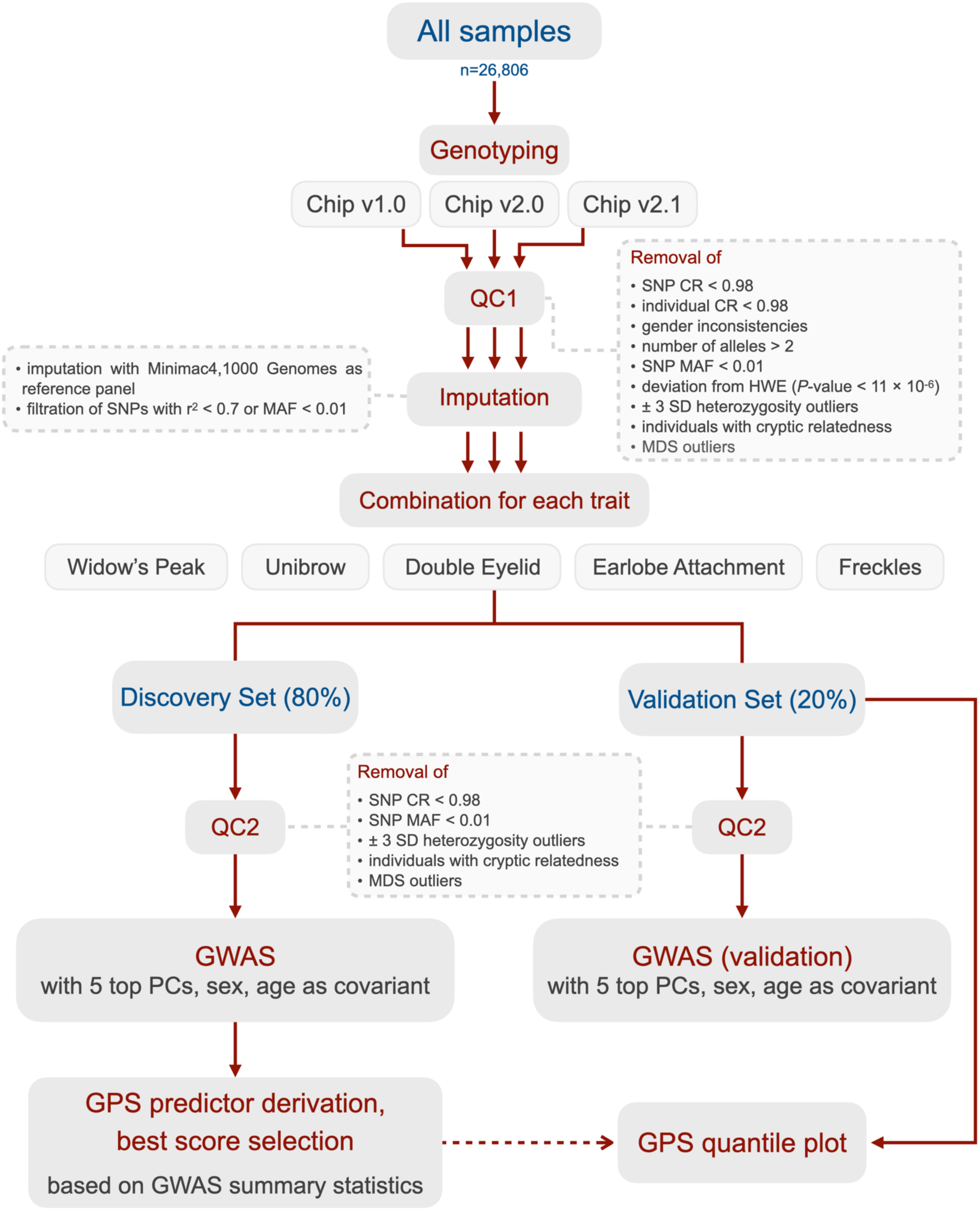


**Supplementary Figure 1.** Flowchart of this study.

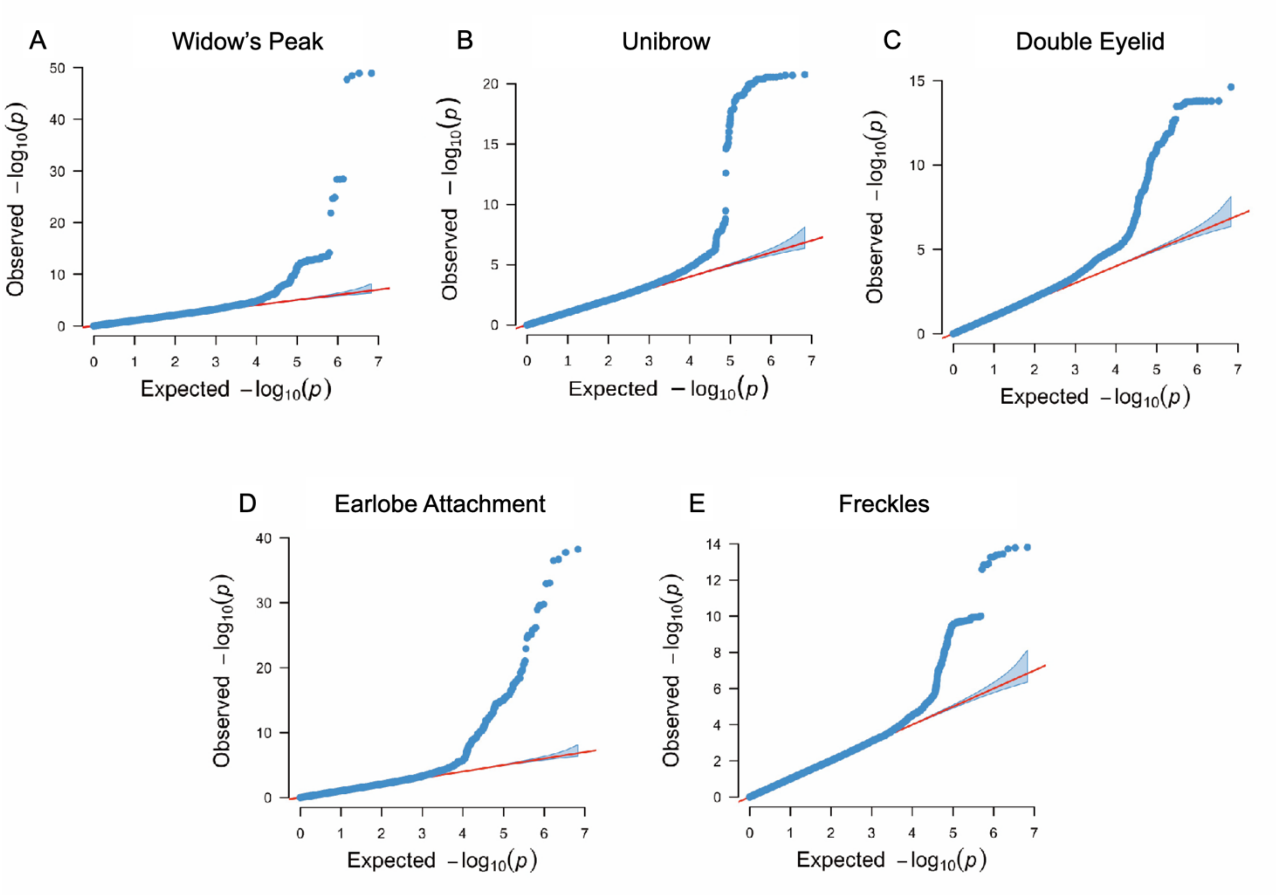


**Supplementary Figure 2.** Quantile-quantile plots for the facial traits. Quantile-quantile plot for (A) widow's peak of observed (lambda=1.04174), (B) unibrow (lambda=1.02955), (C) double eyelid (lambda=1.04324), (D) earlobe attachment (lambda=1.02124), and (E) freckles (lambda=1.00682) *vs*. expected $-\log_{10}(P)$ scores in genome-wide association study (GWAS).

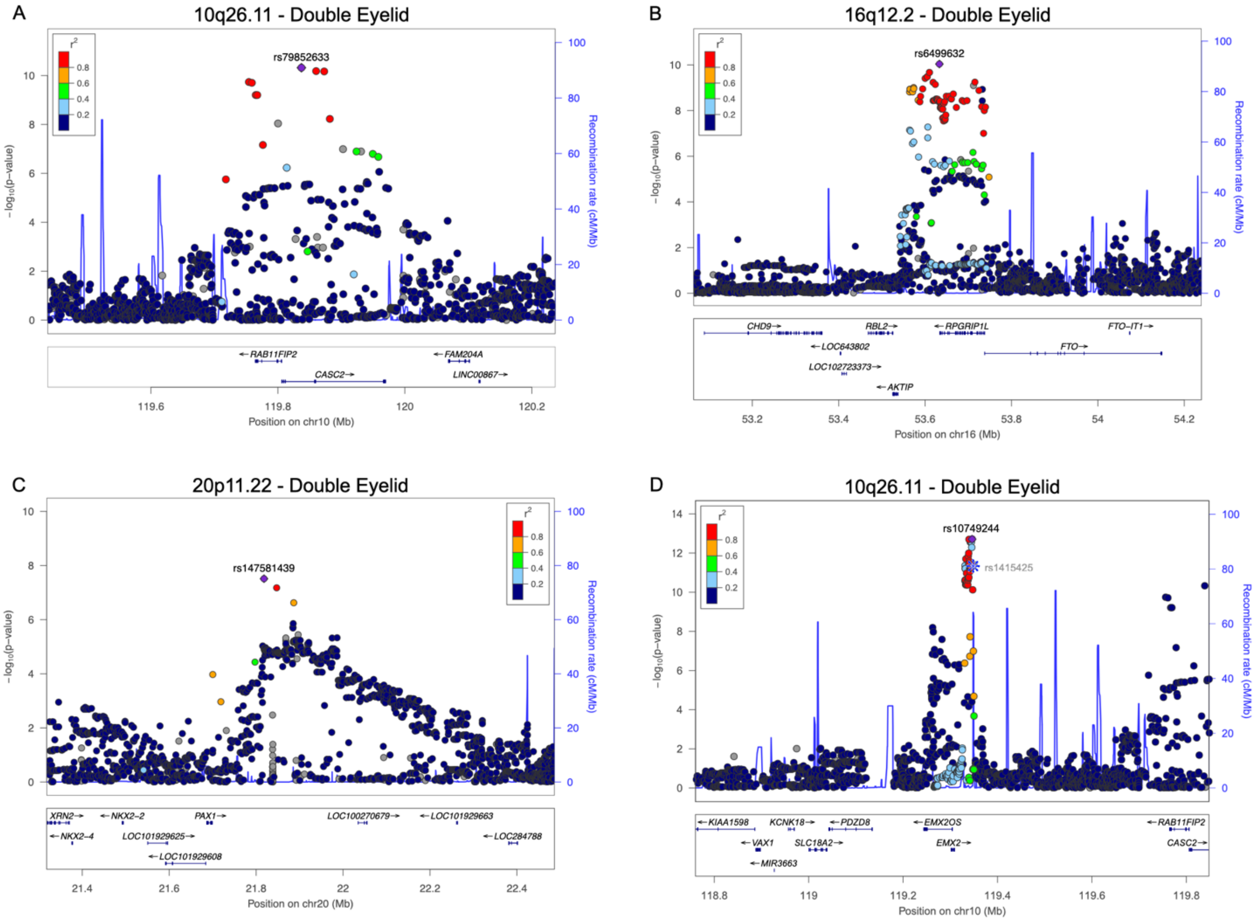


**Supplementary Figure 3.** Regional association plots for four regions of double eyelid associations. Regional association plots for (A) 10q26.11; (B) 16q12.2; (C) 20p11.22; (D) 10q26.11. Reported nearby SNP is marked with snowflake symbol and labeled in grey font.

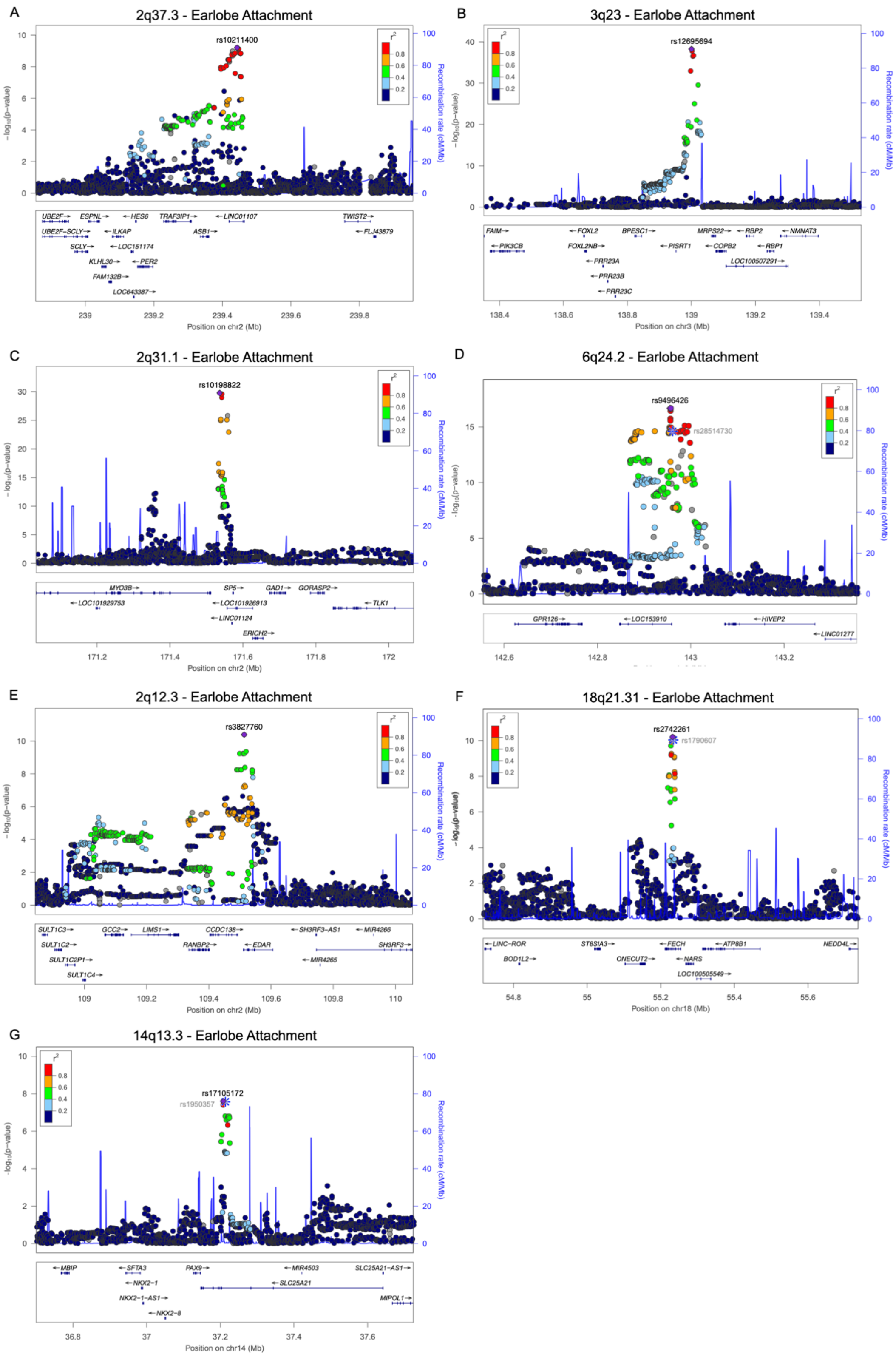


**Supplementary Figure 4.** Regional association plots for seven regions for earlobe attachment associations. Regional association plots for (A) 2q37.3; (B) 3q23; (C) 2q31.1; (D) 6q24.2; (E) 2q12.3; (F) 18q21.31; (G) 14q13.3. Reported nearby SNPs are marked with snowflake symbols and labeled in grey font. SNP, single nucleotide polymorphism.

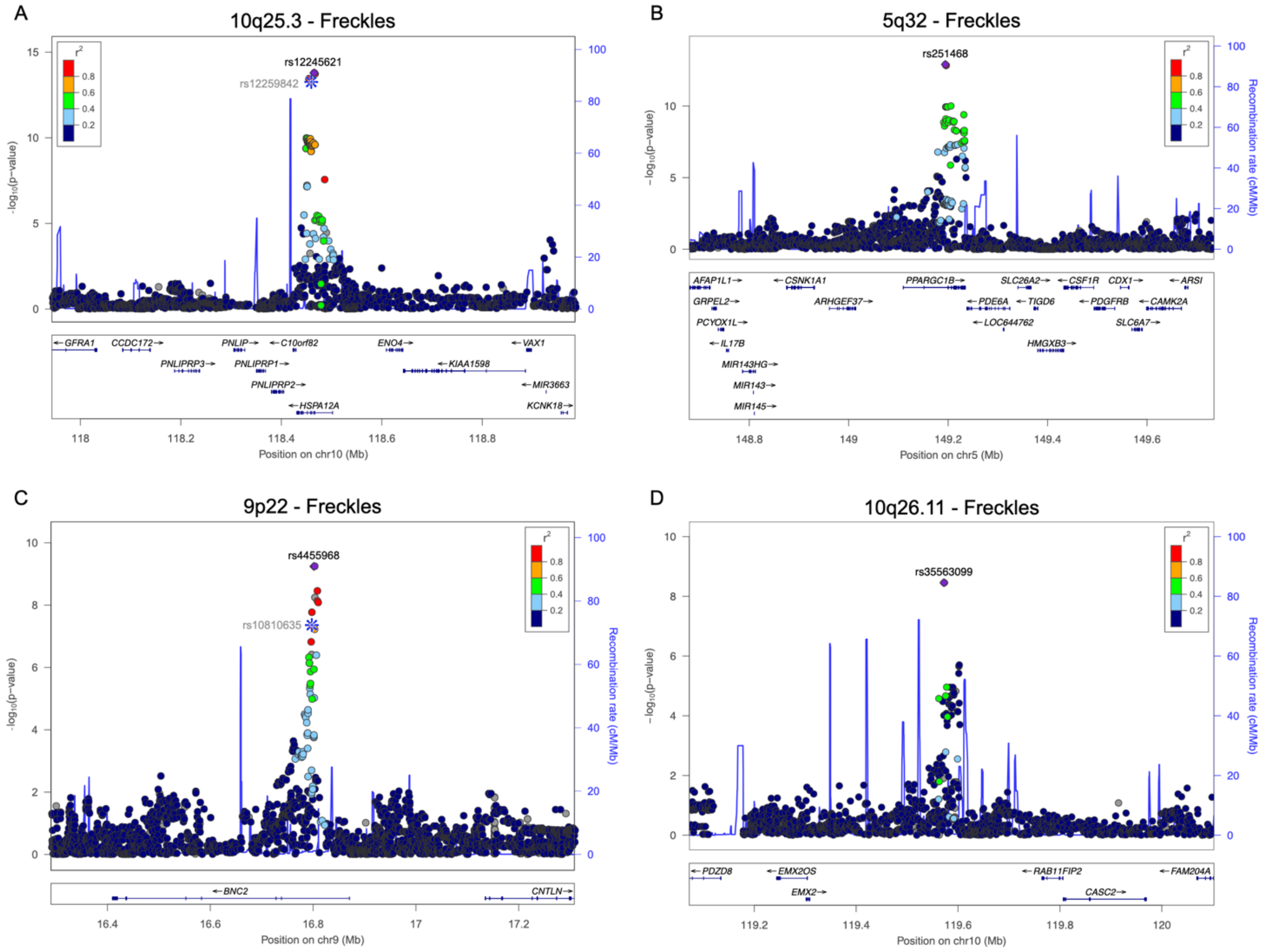


**Supplementary Figure 5.** Regional association plots for four regions with reported SNPs showing genome-wide significant associations with freckles. Regional association plots for (A) 10q25.3; (B) 5q32; (C) 9p22; (D) 10q26.11. Reported nearby SNPs are marked with snowflake symbols and labeled in grey font. SNP, single nucleotide polymorphism.

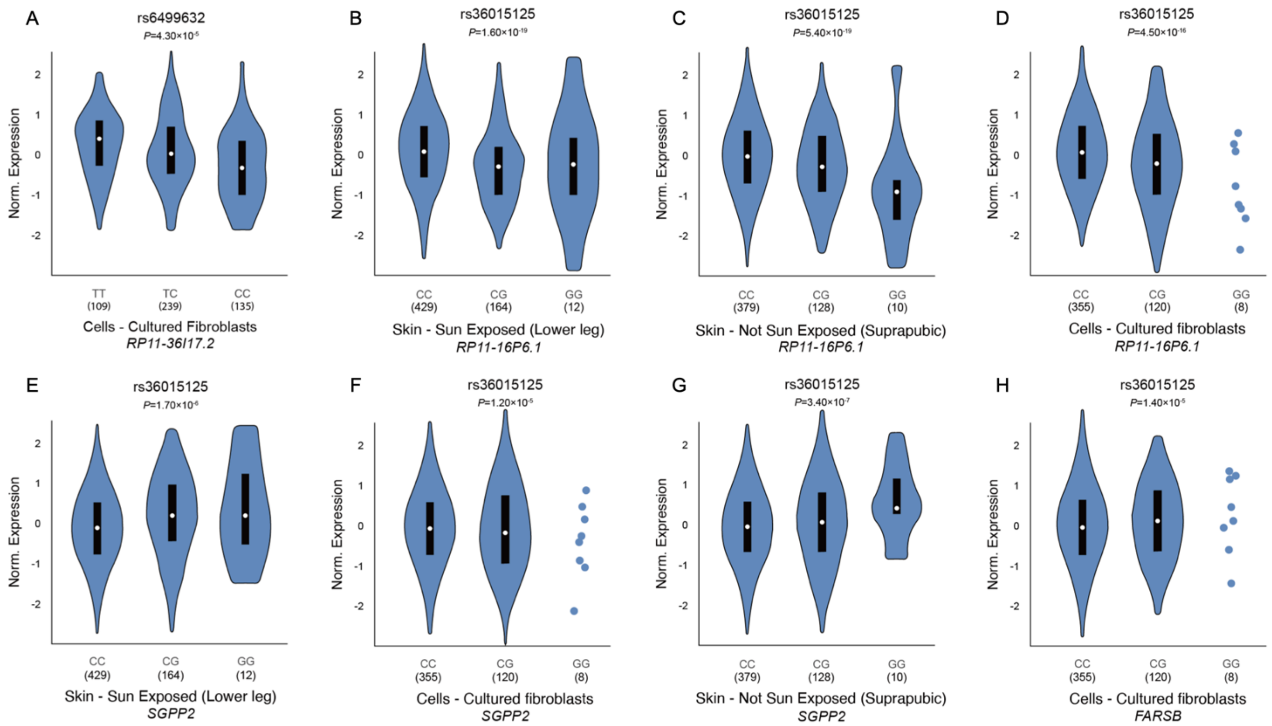


**Supplementary Figure 6.** eQTL violin plot for variants associated with double eyelid and unibrow. Violin plots of eQTL signals for the (A) double eyelid-associated SNP rs6499632 in cultured fibroblasts; (B) unibrow-associated SNP rs3601525 in sun-exposed skin; (C) and (G) unibrow-associated SNP rs3601525 in not-sun-exposed skin; (D), (F), and (H) unibrow-associated SNP rs3601525 in cultured fibroblasts. eQTL information of SNPs reaching genome-wide significance is listed in Supplementary Table 5.

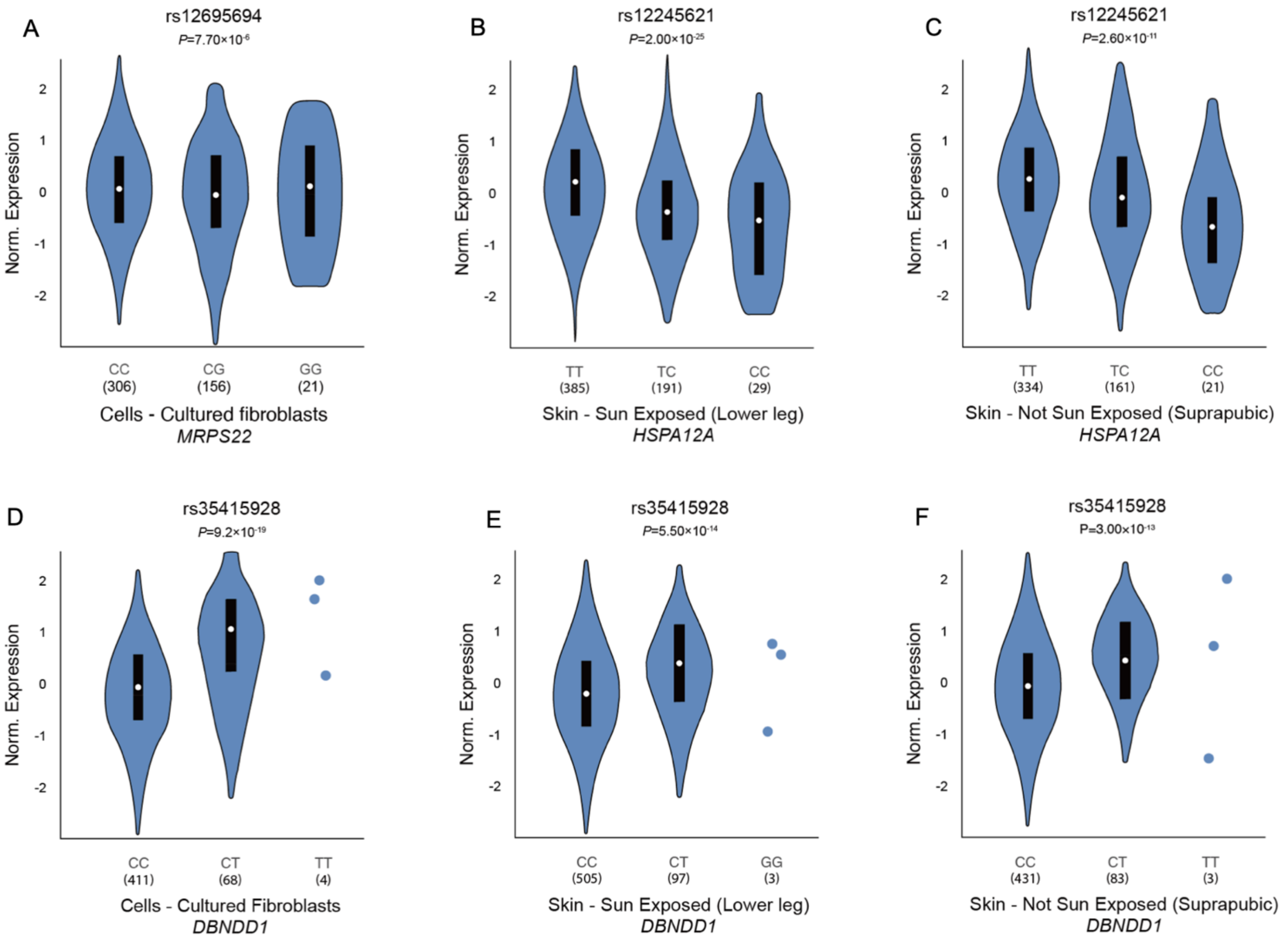


**Supplementary Figure 7.** eQTL violin plot for variants associated with earlobe attachment and freckles. Violin plots of eQTL signals for (A) earlobe attachment-associated SNP rs12695694 in cultured fibroblasts; (B) freckles-associated rs12245621 in sun-exposed skin tissue; (C) freckles-associated rs12245621 in not-sun-exposed skin; (D) cultured fibroblasts; (E) freckles-associated rs35415928 in sun-exposed skin; (F) freckles-associated rs35415928 in not-sun-exposed skin. eQTL, expression quantitative trait locus. Related eQTL information of SNPs reaching genome-wide significance is listed in Supplementary Table 5.
